# Incommensurate Graphene Foam as a High Capacity Lithium Intercalation Anode

**DOI:** 10.1038/srep39944

**Published:** 2017-01-06

**Authors:** Tereza M. Paronyan, Arjun Kumar Thapa, Andriy Sherehiy, Jacek B. Jasinski, John Samuel Dilip Jangam

**Affiliations:** 1 Speed School of Engineering, University of Louisville, 2210 S. Brook st., Louisville, KY, 40208, USA; 2 Conn Center of Renewable Energy Research, University of Louisville, KY, USA; 3 ElectroOptics Research Institute and Nanotechnology Center, University of Louisville, KY, USA; 4 Department of Industrial Engineering, University of Louisville, KY, USA

## Abstract

Graphite’s capacity of intercalating lithium in rechargeable batteries is limited (theoretically, 372 mAh g^−1^) due to low diffusion within commensurately-stacked graphene layers. Graphene foam with highly enriched incommensurately-stacked layers was grown and applied as an active electrode in rechargeable batteries. A 93% incommensurate graphene foam demonstrated a reversible specific capacity of 1,540 mAh g^−1^ with a 75% coulombic efficiency, and an 86% incommensurate sample achieves above 99% coulombic efficiency exhibiting 930 mAh g^−1^ specific capacity. The structural and binding analysis of graphene show that lithium atoms highly intercalate within weakly interacting incommensurately-stacked graphene network, followed by a further flexible rearrangement of layers for a long-term stable cycling. We consider lithium intercalation model for multilayer graphene where capacity varies with N number of layers resulting Li_N+1_C_2N_ stoichiometry. The effective capacity of commonly used carbon-based rechargeable batteries can be significantly improved using incommensurate graphene as an anode material.

Lithium-ion batteries (LIBs) as high energy- and power-density rechargeable batteries are in high demand for energy storage systems. Development of high capacity LIBs anode materials will have a profound and direct impact on current commercial and emerging markets such as portable electronics, electric vehicles and electric grid storage.

Lithium intercalation into graphitic materials has been studied since the 1950’s^1^. Later, it was found that graphite had practical application in rechargeable batteries as an anode material. Graphite-based LiBs exhibit high energy density, high power density, and high current efficiency, and so are commonly employed, though they possess low capacity. Silicon has been recognized as another promising anode candidate for a high-energy density LIBs with a theoretical capacity of 4,200 mAh g^−1^. However, Si anodes exhibit significant volume change during the initial charge/discharge cycles that results in significant chemical and mechanical degradation in anode and consequent rapid capacity fading^2^,^3^. Various scientific and technological solutions have been proposed including to redesign Silicon structures or integrate it with another high conductive protective carbon materials such as graphene^4^,^5^.

The maximum capacity of crystalline graphite (372 mAh g^−1^), consisting of commensurately-stacked layers of graphene, can be achieved by transferring one lithium atom per six carbon atoms resulting LiC_6_ stoichiometry^6^. However, the intercalation occurs at prismatic surfaces (arm-chair and zig-zag faces) only, and no significant lithium reversibly intercalates between commensurately-stacked (referred also as Bernal or *AB* stacking) layers due to the strength of repulsive interactions that arise due to the orthogonality of interplanar *π* orbitals of *sp*^*2*^ carbon. Numerous classes of carbonaceous materials were synthesized over the past 30 years and higher capacities have been reported, but no significant gain in effective capacity has been achieved^7^,^8^. Dahn *et al*. suggested that single layer graphene (SLG) possesses a capacity of 744 mAh g^−1^ on the basis of double-site insertion of lithium as Li_2_C_6_^9^. Sato *et al*. proposed Li_2_ covalent arrangements^10^, where Li atoms intercalate and occupy nearest neighbor sites between each pair of carbon sheets resulting LiC_2_ with 1,116 mAh g^−1^ capacity. Later, Binda *et al*. succeeded in preparing a LiC_2_ compound^11^, but it was unstable in ambient conditions. Indeed, a carbon structure capable of hosting the lithium strongly depends on various factors including crystallinity, surface area, and stacking geometry^12^,^13^,^14^. A high capacity (1,050 mAh g^−1^) was measured for chemically-derived defective graphene^15^, and defect-free few-layer graphene^16^ (850 mAh g^−1^), but these cells were unstable for high discharge rates and for long-term cycling. Recently, the defective porous graphene network demonstrated a tendency to increase capacity during cycling, achieving up to 900 mAh g^−1^ which remained stable over hundreds of cycles^17^. The subsequent calculations confirmed that topologically defective graphene is capable of storing more lithium^18^,^19^. Though, there is not yet a succinct model offering a clear mechanism of lithium intercalation within defect-free multilayer structures or theoretical limit of capacity depend on graphene layers. Multilayer graphene is particularly interesting because its electronic properties can be manipulated through variation of layers and their orientation^20^,^21^. Particularly, the absence of commensurately-stacking order within adjacent graphene layers results in a weaker Van der Waals forces. An increase in rotation angle between layers decreases interplanar interaction so that the incommensurate multilayers can be considered as a single layer with modified electronic structure^22^,^23^,^24^. In fact, the incommensurately-stacked infinite layer graphene can be considered “graphite-like” structure with weakened interplanar interaction exhibiting novel physical and electronic properties. It has been shown that theoretical quantum capacitance of multilayer graphene significantly improves by altering its local structure and morphological features^25^.

In this study we demonstrate a novel incommensurately-stacked graphene 3D network and its use as a high-capacity lithium intercalation anode in rechargeable batteries. We found that specific capacity is enhanced when commensurately stacking order of graphene layers was reduced in an anode material. As-grown highly (up to 93%) incommensurate graphene demonstrates four times higher reversible capacity (1,540 mAh g^−1^) than theoretical capacity of graphite which is stable throughout 100 cycles. Testing for 100 charge/discharge cycles 86% sample indicates excellent reversibility with over 99% coulombic efficiency with 930 mAh g^−1^ capacity. To explain exceptionally high capacities, we investigate lithium intercalated graphene structures and propose a model in which the finite layers of graphene can demonstrate higher capacity, achieving a maximum of 1,674 mAh g^−1^ in bilayer configuration.

## Results and Discussion

**Figure 1:**
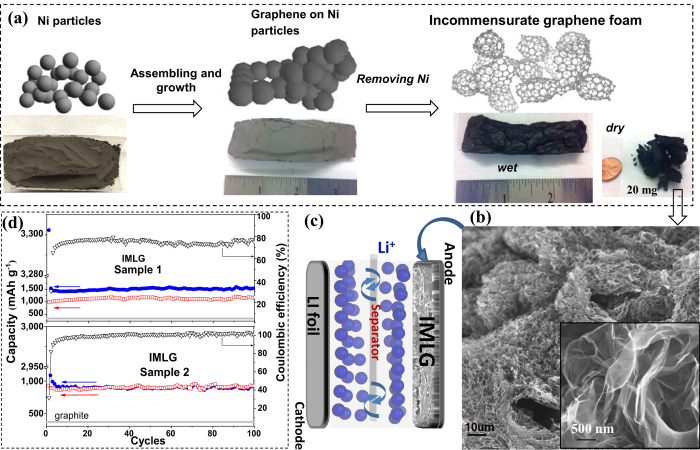
Illustration of the preparation route of incommensurate graphene foam and battery testing. (**a**) Schematic illustration of graphene foam preparation. (**b**) Scanning Electron Microscope (SEM) image of graphene foam. (inset)- low scale image of a foam. (**c**) Schematic construction of battery cells. (**d**) Capacity cycling for Sample 1 and Sample 2 tested at 100  mA  g^−1^: *blue dots* - discharge*, red squares* - charge capacity, *black open triangles* - coulombic efficiencies (axis in right).

**Figure 2:**
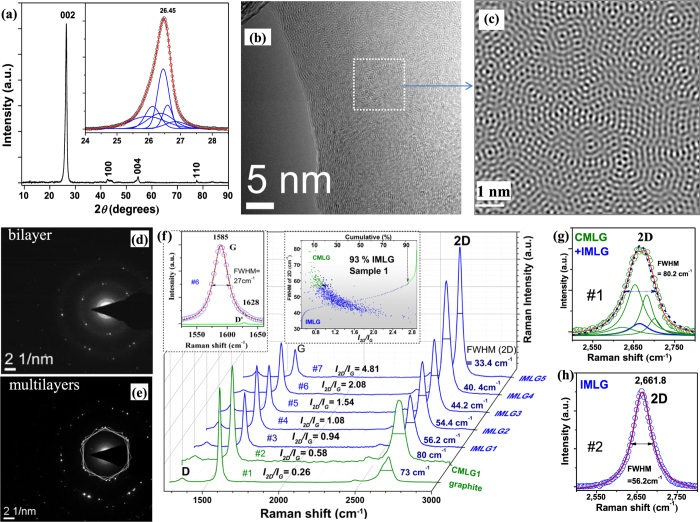
Characterization of incommensurate pristine graphene foam. (**a**) XRD pattern of graphene foam. (inset)- Gaussian fit of (002) peak. (**b**) HRTEM image of graphene sheets. (**c**) FFT pattern of a masked area in the image (**b**). (**d**) SAED pattern of bilayer rotated at 5°. (**e**) SAED pattern of many-layer sheets: five single hexagonal patterns drawn to demonstrate the rotations 5–30°. (**f**) Individual Raman spectra (638  nm laser wavelength) of *incommensurate* (spectra # 2–7) and *commensurate* (spectrum *#* 1,2) graphene. (inset in middle)- Scattergram of FWHM of 2D and *I*_*2D*_*/I*_*G*_ for Sample 1 analyzed for 960 spots by 5 × 5 (X;Y) μm step of Raman mapping. IMLG (*blue squares*), CMLG (*green dots*) and (*dark blue)-* (2% %) the spots when single- and multi-Lorentzian fits equally, (inset in left)-Lorenztian fit of G band of spectra #7. (**g**) Multi- and Single- (**h**) Lorentzian fits of 2D peaks presented in (**f**).

In recent years, copper and nickel have been found to be an excellent catalytical substrates to develop two^26^,^27^,^28^ and three dimensional (3D) commensurate multilayer graphene (CMLG)^29^,^30^,^31^ for various applications. Here, we grew a thin graphene film on few-micron sized nickel particles, assembled as 3D template, by decomposition of methane at 1,000–1,050 °C under low pressure (Fig. 1a and see Supplementary Fig. 1). After etching the nickel, only graphene network remains which consists of micron-sized curved transparent sheets connected to each other as a foam without significant damage and agglomeration and which are separated from each other by micro-pores (Fig. 1b, Supplementary Figs 2 and 3). The curvature of graphene sheets, presumably, originates from the catalyst shape since there is similarity in their morphology. A relatively large amount (~20 mg) incommensurate multilayer graphene (IMLG) 3D network was collected from each process which was enough to fashion into an anode material for many coin cells (Fig. 1c). The final graphene product contains 97–99% carbon with very low oxygen content (see Supplementary Fig. 4). X-ray photoelectron spectroscopy (XPS) shows that the carbon is primarily *sp*^2^ with relatively low amounts of *sp*^3^ and C = O components and no significant amorphous carbon (see Supplementary Figs 5 and 6). X-ray diffraction (XRD) pattern shows a sharp (002) peak at 2*θ* = 26.45° corresponding to the average *d* layer spacing of 3.36 Å and accompanied small (004) peak at 54.73° (Fig. 2a). These peaks indicate interplanar stacking, while the absence of the other diffraction peaks such as (101), (112), (113) *etc*. indicates the absence of commensurate stacking within layers. The (100) and (110) peaks at 42.63° and 77.57°, respectively, originates from in-plane crystallinity of graphene. The Gaussian fit of the (002) peak reveals few peaks with *d* varies from 3.34 to 3.45 Å as an evident of various number of layers (inset- Fig. 2a) similar to graphite. Specific Surface Area (SSA) of graphene foams shows 90–100 m^2^g^−1^ as measured by Bruner-Emmett-Teller (BET) method (see Supplementary Fig. 7), which is 26–30 × less of SLG (theoretically 2,630 m^2^g^−1^) assuming these graphene samples consists of 26–30 layers on average. Similarity of *d*-spacing suggests that no Nitrogen gas absorbs between layers, and an increased SSA is the result of thinner graphene sheets with pores.

High-Resolution Transmission Electron Microscopy (HRTEM) confirms that the sheets contain 2–30 layers (see Supplementary Fig. 8), and much thicker (up to 50 layers) sheets present only occasionally. Fast Fourier Transform (FFT) filtering of HRTEM image (Fig. 2b) results in Moiré patterns (Fig. 2c), which are observed when layers are rotated or twisted^32^. Selected Area Electron Diffraction (SAED) measurements, under TEM, show diffractions of two or more hexagonal single-crystalline patterns rotated at 5–30° angles (Fig. 2d,e and Supplementary Fig. 9), which indicate the misorientation of layers.

Raman spectra provide the structural and electronic details of graphene-related materials such as stacking order, the number of layers, quantity and nature of defects, and doping^33^,^34^. The shape and width of the 2D band can distinguish the stacking^35^,^36^ and relative orientations^37^. Multilayer graphene 2D band either is single- or multi- Lorentzian fit discriminates between commensurate and incommensurate stacking (Fig. 2f–h). The values of 2D bandwidth (presented as full width of half maxima -FWHM) and *I*_*2D*_*/I*_*G*_ (here, *I*_*2D*_ and *I*_*G*_ are the heights of 2D and G bands, respectively) of forty-five typical Raman spectra from various regions of samples were carefully analyzed (see Supplementary Table 1) to classify *incommensurate* and *commensurate* stacking. The higher is the ratio *I*_*2D*_*/I*_*G*_ and narrower 2D band is, the weaker the interplanar coupling is, thus closer resembling to spectrum of SLG. However, we found both 2D and G bandwidths are broader than we expect for SLG, even *I*_*2D*_*/I*_*G*_ = 4.81 confirming this graphene is not a single layer.

A direct determination of FWHM and *I*_*2D*_*/I*_*G*_ values can also be acquired to generate Raman maps. Thus, we analyzed 2D and G bands by processing several maps to estimate percent of incommensurate stacking in graphene foam (see Supplementary text and Supplementary Table 2). Aberration of these values of incommensurately stacking spots is likely caused by the variety of interplanar rotation angles of adjacent layers as noted in ref. 37. The 2D band becomes broader and consists of multi- Lorentzian of a mixture *commensurate* and *incommensurate* due to their overlapping exposure to laser^38^ which we still count as CMLG (Fig. 2g).

Sample 1 was estimated 93% of IMLG (inset- Fig. 2f), which often exhibits impressive homogeneity in the large mapping areas (Supplementary Fig. 10). The position of 2D peak does not show significant variation overall. It is important to note that FWHM in the range of 56–65 cm^−1^ with *I*_*2D*_*/I*_*G*_ ≥ 0.94 (Fig. 2f) 2D peak has a similar fit to either single- or multi- Lorentzian (~2% of the 960 spectra) which we also classify as IMLG. Another six samples were estimated 19–86% of incommensurateness (referred as Sample 2–7 or S2-S7) by analysis of 250–760 spots of several regions for each sample and tested as an active electrode in LiBs. Though, we can suggest that small atoms/ions such as lithium may easy penetrate within layers due to weakened interplanar interaction.

High crystallinity determines the charge collection in electrodes since the electrical conductivity arises in part from the hopping of carriers between crystallites. *L*_*a*_ crystallite size and defect concentration was estimated by *I*_*D*_*/I*_*G*_ (here, *I*_*D*_ is the height of D band)^39^,^40^ *L*_*a*_ = 467.8–568 nm which confirms high crystallinity in-plane (Supplementary text and Supplementary Fig. 10c). Raman mapping analysis (Supplementary Fig. 10d) revealed that *I*_*D*_*/I*_*D′*_ (here*, I*_*D*_′ is the intensity of D’ band presented left inset in Fig. 2) values were in the range of 3.3–7.5 (Fig. 2e) which can be assigned to the boundary defects^41^. Very small amount of values (less than 10%) are in the range of *I*_*D*_*/I*_*D*_′ = 7.5–11.5 which can be assigned to vacancies such as topological defects and carbon voids. Overall, low defect concentration (~ 0.02% of carbon) is similar for all seven graphene samples which allowed us to neglect any impact of original defects on the battery performance.

**Figure 3:**
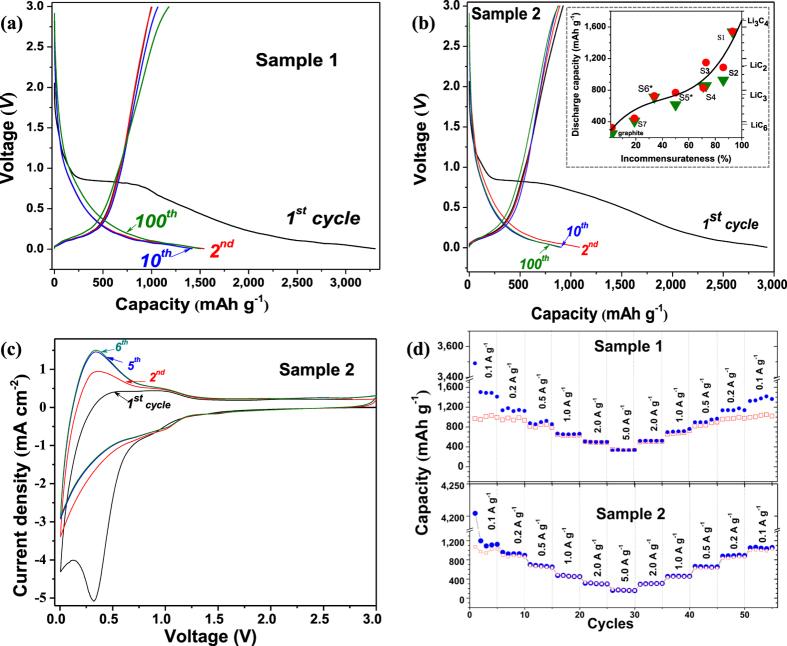
Electrochemical characterization of IMLG-based coin cells. (**a**) Charge-discharge voltage vs. specific capacity (at 100  mA  g^−1^): 1^st^ (*black*), 2^nd^ (*red*), 10^th^ (*blue*) and 100^th^ (*green)* cycles for Sample 1 and Sample 2 (**b**). (inset) in (b) shows the plot of discharge capacities versus incommensurateness of pristine graphene samples; *red dots* -second cycle, *green triangles* - 100^th^ cycle (80^th^ cycles indicated by asterisks). The curve presents polynomial fit of 100^th^ capacity. (**c**) CV curves of Sample 2 at 3.0–0.005  V with a scan speed of 1mVs^−1^. (**d**) *C*-rate testing for Sample 1 and 2 at different current densities: *blue dots* - discharge*, red squares* - charge capacities.

Graphene foams (~1 mg) with 19–93% incommensurateness (Samples 1–7) were prepared as thin electrodes and tested over 80–100 cycles in electrochemical cells (EC) (Fig. 3 and Supplementary Fig. 11). Voltage-charge/discharge measurements (Fig. 3a,b) show that initial discharge (Li-insertion) capacity profiles are different from the subsequent cycles caused by formation of a passivating film (referred to as the solid electrolyte interface- SEI). The second discharge capacity was increased from 444 mAh g^−1^ (Sample 7) up to 1,542 mAh g^−1^ (Sample 1) by an increase in graphene incommensurateness from 19% up to 93% (Supplementary Table 3). Commercial graphite was also tested which performs 258 mAh g^−1^ capacity at 100^th^ cycle. Discharge capacity shows nonlinear dependence on incommensurateness (inset-Fig. 3b) with the Li_3_C_4_ stoichiometry maximum corresponding to capacity of 1,674 mAh g^−1^. We assume that other factors such as interplanar rotation angle and number of layers affect to the formation of reversible capacity. The reversible capacity stabilized within the first 3–5 cycles, and charge-discharge retention remained over 95% throughout 100 cycles. Thus, at least five charge-discharge cycles were run for further characterizations of electrodes. The cells that exhibit 1,030–1,100 mAh g^−1^ at the second discharge cycle (as Sample 2 in Fig. 1d) demonstrate high coulombic efficiency up to 100% after a few cycles. The cells that exhibit a higher capacity than 1,100 mAh g^-1^ in the 2^nd^ discharge cycle demonstrate lower (73–75%) coulombic efficiency (as Sample 1 in Fig. 1d). A larger amount of lithium has been inserted at initial intercalation stage of Sample 1 (3,302 mAh g^−1^) than for Sample 2 (2,933 mAh g^−1^) even though there is no significant difference in incommensurateness, defect’s concentration and BET results between these two samples (100.8 m^2^g^−1^ SSA for Sample 1 and 95.7 m^2^g^−1^ SSA for Sample 2). Therefore, we consider an additional factor, the number of graphene layers, to explain this variance in capacity. Recombination of layers during the second cycle may govern further reversible capacity and coulombic efficiency as well on subsequent cycles. The incommensurately-stacking feature of pristine layers such as rotation angle and interaction with commensurate layers can play significant role in the initial intercalation stage, and lead to further reconstruction of layers.

The rate capability of the cells and Li/Li^+^potential were characterized by cyclic voltammetry (Fig. 3c). The shape of curves matches the voltage plateaus of profiles in Fig. 3b. The anodic peak at +0.34 V is fully developed and stabilized within first 5 cycles indicating maximum lithium insertion. Figure 3c shows *C*-rate testing exhibits stability of the cells under high current densities which makes these cells feasible.

As the measured reversible capacities are considerably high, at that point we consider a lithium insertion model other than a model of LiC_2_ by Sato *et al*., ref. 10, which would explain any LiC_2−x_ (0 ≤ x ≤ 2) formation. In this regard, we prepared electrodes and investigated the structural, binding and electronic changes of graphene after five cycles of battery tests.

**Figure 4:**
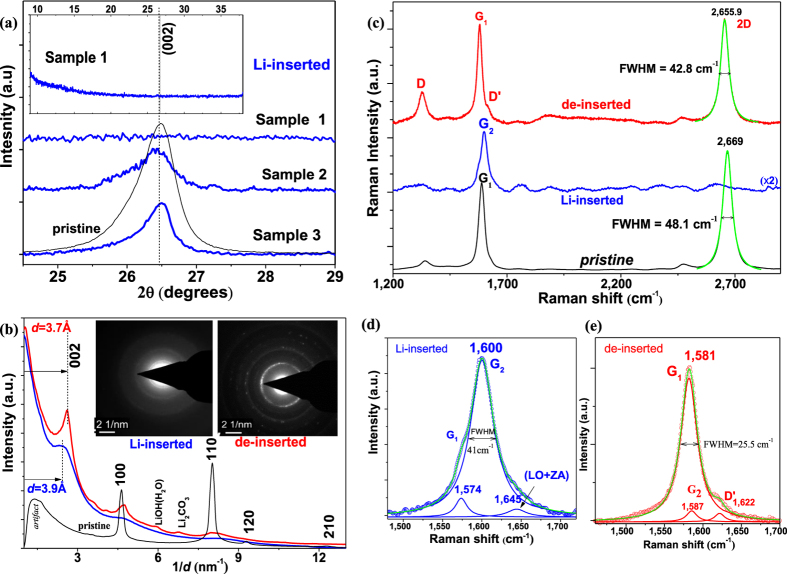
Structural and electronic characterization of IMLG-electrodes. (**a**) XRD (002) peaks of lithiated electrodes of Sample 1–3. The baseline subtracted similar way. (inset)- XRD of Sample 1 at 9–38°. (**b**) The plots of intensity vs. inverse distance based on SAED of Sample 2 nanosheets: *blue-*Li-inserted, *red*- de-inserted, *black -* pristine. SAED patterns of Li-inserted (*left inset*) and de-inserted (*right inset*) sheets. (**c**) Raman (λ = 638  nm) spectra of unexposed electrodes of Sample 2 after 5^th^ cycle: an averaged 80 spots (mapping by 5 × 5 (X; Y) μm) of Li- inserted (*blue curve*) and de-inserted (*red curve*). Lorentzian fits of 2D bands is highlighted. Lorentzian fit of G bands for Li-inserted (**d**) and de-inserted (**e**) of spectra presented in (**c**).

The XRD (002) peak of intercalated electrodes (unexposed) is lower and broader than pristine samples as measured after 5^th^ cycle (Fig. 4a). This type of (002) peak with smaller (100), (004) and (110) peaks observed for the electrodes which demonstrated 800–850 mAh g^−1^ specific capacity as Sample 3. Note that this peak was decreased (as Sample 2) with increased capacity and it disappeared from its nominal position (2*θ* = 26.45°) when capacity was greater of 930 mAh g^−1^ (as Sample 1). De-inserted electrodes exhibited similar tendency with the same capacities. When lithium intercalates within commensurately-stacked graphene layers such as graphite, the sheets rotate from *AB (d* = 3.35 Å) into *AA* stacking (*d* = 3.6 Å) bringing carbon honeycombs directly above and below one another^42^. Thus, for intercalated electrodes, the (002) peak is expected shift to lower 2*θ* position. On the contrary, no noticeable Bragg peak was observed within 38° and 9° (inset- Fig. 4a) for highly intercalated samples. The absence of (002) peak could be result of single layers interacted with larger *d-*spacing (*d* ≥ 3.6 Å)^43^ by elimination of interplanar interaction or significant damage of structure which can be confirmed by Raman analysis.

SAED was measured for several sheets which indicates that the stacking geometry of incommensurate graphene sheets changes after the insertion/de-insertion of lithium. The revealed *d*-spacing peaks at *d*_Li-inserted_ = 3.9–4.06 Å and *d*_de-inserted_ = 3.65–3.8 Å (Fig. 4b) indicate *AA* stacking. As we can see, the lithium is adsorbed between two layers at the distance of 1.85–2.03 Å (1/2 *d*_*Li*_*−*_*inserted*_) which correlates with the theoretically calculated values of lithium hosting distance from graphene1.84–2.02 Å^44^. We conclude that in the initial cycle a large amount of lithium atoms adsorb into graphene layers due to their weaker interactions and moves graphene layers farther apart resulting in free expansion of the structure either inner or outer sites of sheets. Once the sheets were adjusted to *AA* stacking during lithium intercalation, they stayed in that position after de-insertion governing further reversible cycling. This reorganization is different from the intercalation of graphite, where the sheets return back to *AB* initial stacking during de-insertion.

Structural and electronic changes in the graphene were obvious also by Raman analysis of electrodes (Fig. 4c–e and Supplementary Fig. 12). The shift and splitting of G band was observed: G_1_- related to interior carbon layer with no lithium intercalated and G_2_- related to bonded carbon layer with lithium^45^,^46^. The height ratio of G_1_ to G_2_ peaks was 0.08 averaged (Fig. 4d) for 80 spectra (Supplementary Fig. 13) by mapping of Sample 1 which increases to 14.7 after de-insertion (Fig. 4e) and confirms that lithium atoms are easy reversible bonded/de-bonded with *sp*^*2*^ carbon. The peak at 1,645 cm^−1^ is associated with the out-of-plane LO + ZA phonon mode^47^ and here is probably caused by bonding of lithium. The 2D and D bands nearly disappeared due to lithium insertion which is similar to highly intercalated graphite^48^. De-inserted samples after 100 cycles performed great homogeneity in ratio of G and 2D peaks (Supplementary Fig. 14) and no significant change of D band for both low and high capacity demonstrated samples. The slight increase in D band is related to small amount of additional boundary defects (estimated by *I*_*D*_*/I*_*D’*_values in Supplementary Fig. 14e). Usually, the perturbation caused by the intercalated lithium atoms shouldn’t have noticeable contribution to Raman D and D’ bands^49^, and a slightly increased D band can be the result of the possible oxidation reactions of the residual lithium on the graphene edges. Single-Lorentzian is always good fit into 2D band (Fig. 4c), and the broad variation of *I*_*2D*_*/I*_*G*_ and FWHM for pristine samples became narrower (Supplementary Fig. 14c,d) which indicates homogeneity of the structure in both in-plane and out-of-plane.

Thus, Raman analysis confirms that structural change out-of-plane has occurred due to intercalation but no significant damage in-plane happened after long-term cycling. The absence of the (002) Bragg peak is rather caused by delamination of multilayer graphene into single sheets than in-plane structural damage.

Usually, when lithium atoms adsorbs onto a carbon structure, it stabilizes by weakly binding its 2 *s* electron with carbon 2*p* orbitals. When transferred lithium atoms are not fully involved in charge transfer, the battery lifetime reduces due to metallic lithium plating the anodes.

**Figure 5:**
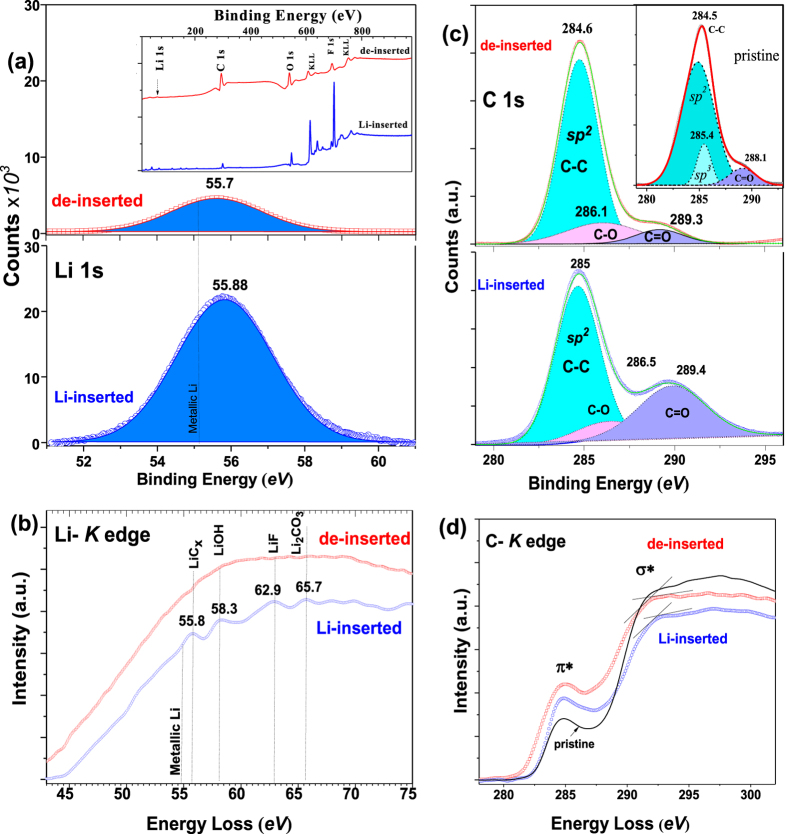
Lithium-carbon binding analysis of Sample 1. (**a**) XPS spectra of Li 1 s for Li-inserted and de-inserted electrodes: (inset)- XPS survey. (**b**) Li-K edge by EELS for Li-inserted *(blue*) and de-inserted (*red*) nanosheets. (**c**) XPS spectra of C 1 s. (inset)- C-K edge of pristine graphene. (**d**) C-K edge of the Li-inserted (*blue*) and de-inserted (*red*) nanosheets.

The Li 1 s has been registered at 56 eV by XPS for LiC_2_ and it tends to the decrease towards to the metallic Lithium (55.2 eV) with increase of Li concentration^50^. Sample 1 performs single- Gaussian 1 s peak of lithium at 55.88 eV (Fig. 5a) which can be assigned to LiC_2−x_ (0 ≤ x ≤ 2). Electron Energy Loss Spectrometry (EELS) analysis of Li-inserted nanosheets, under TEM, revealed Li-K edge at 55.8 eV (Fig. 5b), which is considerably lower than for LiC_6_ (57.5 eV)^51^. However, no metallic (54.97 eV) Lithium or other oxidized species of metallic Lithium (Li_2_O, Li_2_O_2_) were revealed by either XPS (~56.5 eV) or EELS and most lithium atoms transferred to graphene participated in charge transfer. Note that samples were briefly exposed to air during transfer to the vacuum chambers. The peak at 58.3 eV on EELS is assigned to LiOH as a result of reaction between lithium atoms and H_2_O.

The XPS spectrum of C 1 s shows multiple splitting in Gaussian fit by forming C-C (*sp*^*2*^), O-C-O and C = O bonds (Fig. 5c). The shift of *sp*^*2*^ peak by 0.5 eV (compared to pristine) confirms the charge transfer to carbon. The C = O peak associated with Li_2_CO_3_ can arise from the reaction of Li^ + ^with carbonates of the electrolyte or with the reaction in air. Sufficient decrease of C = O peak for de-inserted samples indicates that most lithium was de-bonded during the de-insertion as shown in Fig. 5a. Carbon K-edge by EELS shows no chemical shift in the π* resonance, but there is a shift to the higher energy (0.6 eV) in the onsets of σ* orbital caused by charge transfer (Fig. 5d). Thus, we conclude that high capacity is caused by large amount of charge transfer of Li^ + ^ions and insertion mechanism occurs other than reported so far. Sato’s mechanism considered maximal capacity of 1,116 mAh g^−1^ according to LiC_2_ formation when Lithium atoms can form Li-Li dimer molecule due to weakly binding of Li 2 s electrons on carbon site. Here, XPS analysis revealed that electrodes demonstrated large amount of Li 1 s electrons interacted to Carbon, and no metallic Lithium was registered either XPS or EELS which allow us to consider ionic lithium (Li^ + ^) model.

To confirm consistency of our EC measurements, we estimated Li:C ratio by appropriate interpretation of Li 1 *s* and C 1 *s* peaks (dividing by sensitivity factors). An averaged LiC_1.83_ (capacity of 1,220 mAh g^−1^) was acquired from various spots of the sample which demonstrated a capacity of 1,269 mAh g^−1^ at the 5^th^ cycle of EC measurement.

**Figure 6:**
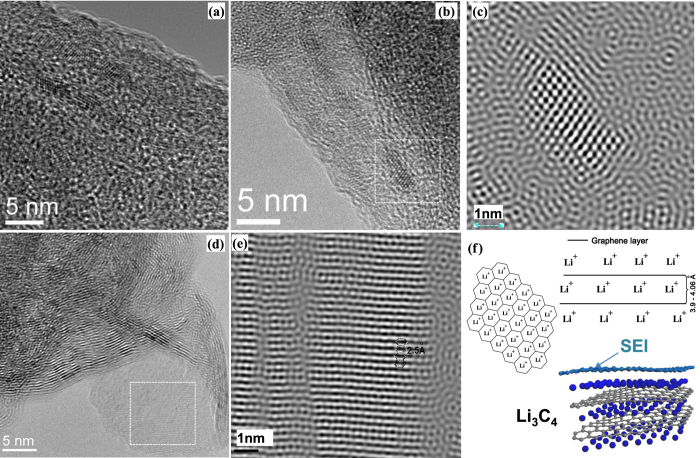
HRTEM images of graphene electrodes and intercalation mechanism. (**a**), (**b**) HRTEM images of Li-inserted sheets of Sample 1 after the 5^th^ cycle. (**c**) FFT filtering of masked area in (**b**). (**d**) HRTEM image of de-inserted graphene sheets of Sample 1 after 5^th^ cycle. (**e**) FFT filtering of the masked area in image (**d**). (**f**) Schematic illustration of lithium insertion in multilayer graphene.

HRTEM imaging of intercalated nanosheets detached from Sample 1 revealed well-aligned periodic patterns with respect to graphene planes (Fig. 6a–c). The measured interatomic spaces are varied from 3.08 to 3.3 Å. Though, these values are larger than spaces between centers of hexagons (2.46 Å), where we most expect engagement of lithium atoms, but it is smaller than metallic Lithium-3.51 Å. The efforts to find similar periodic patterns on de-inserted or pristine nanosheets were unsuccessful. De-inserted nanosheets are noticeable different of Li-inserted (Fig. 6d and Supplementary Fig. 15) and the linear patterns were observed everywhere. FFT analyses revealed interline distances of 2.5–2.6 Å (Fig. 6e) similar to the distances of carbon bonds in hexagons (2.46 Å) which can result due to *AA* stacking. Moiré patterns are observable for small regions as an evidence of uncompleted *AA* stacking (Fig. 6c,e).

We attempted to define the nature of observed periodical patterns of intercalated samples by conducting to EELS measurements of the area which confirms the failure to detect metallic Lithium, but LiOH along other lithium species (Fig. 5b) were revealed as indicative of lithium atoms and ions. The arrangement and periodicity of those patterns led us to assign it to lithium atoms in manner as every atom was associated to each hexagonal ring of graphene (Fig. 6f). The binding of OH- groups to lithium atoms as a LiOH may cause interatomic extension on HRTEM image but we also consider that interatomic spaces can be affected by other reasons such underneath Li layers, the rotation angle of adjacent graphene layers, in-plane graphene curvature and Li-Li repulsive forces. However, *in-situ* HRTEM measurements and further calculations are necessary for complete understanding the intercalation mechanism within incommensurately-stacked graphene layers.

Thus, based on XRD structural analysis of intercalated samples we conclude that all galleries of incommensurately-interacted graphene sheets are fully occupied by lithium and they participate in charge transfer as Li^ + ^. In addition to structural and binding changes of lithiated graphene HRTEM observations allow us to propose a model where lithium atoms adsorb above and below each hexagon in multilayer configuration (Fig. 6f). Then Li:C ratio would vary by N number of layers resulting Li_N*+*1_C_2N_ stoichiometry (Supplementary text and Supplementary Table 4). The capacity reaches maximum (1,674 mAh g^−1^) when bilayer participates in charge transfer as Li_3_C_4_ while an infinite number of N would approach to LiC_2_ (1,116 mAh g^−1^) (Supplementary Table 4 and Supplementary Fig. 16).

The larger number of initial layers can lead to higher capacity of the cells if interplanar interaction forces are weak enough to host larger amount of lithium atoms within graphene layers during the initial intercalation process and split them much longer distances due to lithium accumulation or Li-Li repulsive forces. As we found by XRD/SAED results, during de-insertion process the layers do not return back to initial *d*-spacing position and graphene interplanar interaction became even weaker than in pristine layers (absence of XRD 002 peak). Then, during the re-intercalation at the second cycle, the recombination of layers can happen differently by coupling of various number of graphene layers (Supplementary Fig. 17). In fact, highly incommensurateness degree with larger crystallites can lead to the best intercalation and high flexibility of layer arrangements.

## Conclusion

We successfully developed graphene foam with up to 93% of incommensurately-stacked multilayers that shows four times higher capacity of storing lithium than theoretical capacity of graphite. Large in-plane crystallites were produced over well-interconnected curved graphene sheets with separation of few-micron size pores. This graphene network demonstrates a reversible capacity of 1,540 mAh g^−1^ (75% coulombic efficiency) and 930 mAh g^−1^ (over 99% coulombic efficiency) applied as an anode in lithium battery cells which was stable throughout hundred cycles at the high current density. We propose a lithium insertion model for multilayer configuration where all carbon hexagons are occupied by lithium atoms and the specific capacity increases as the number of graphene layers decreases. We found that high crystallinity and high incommensurateness within layers are key factors for exceptionally increased capacity. In fact, the weakened Van der Waals interaction of graphene layers enables easy and full penetration of lithium atoms, followed by flexible adjustment of the layers for stable cycling. An effective capacity increase over six times compared to traditional commercial graphite cells promises the feasibility of the rapid development of lightweight, cost-efficient, high-capacity rechargeable batteries based on the work presented here.

## Methods

### Incommensurate Multilayer Graphene (IMLG) foam preparation

Commercially-available Ni powder with 1–15 μm size of particles (99.9% metal basis -Alfa Aesar) was used as a catalyst and substrate to grow graphene. The catalyst particles were placed into a 1.5″ diameter quartz tube and heated up to 1,000 °C ~50 mTorr of pressure using Ar/H_2_ (3:2) (Research grade) gas mixture ~80 sccm rate as a carrier gas. A relatively low rate of methane gas, e.g. 5–8 sccm, was applied as a carbon source at 1,000–1,050 °C, and then the furnace was cooled down to room temperature at 100 °C/min rate. The nickel was then separated from the graphene by etching of Ni/graphene template directly in acids (1 M HCl + 1 M HNO_3_) followed by washing of the residue in DI-water several times. Large black pieces flooded the water surface once the Ni metal template was removed completely. To avoid agglomeration in the graphene network, a drying process was then carried out using a Critical Point Dryer (CPD) (Samdri® PVT-3D). The graphene network was rinsed with pure ethylene alcohol several times by replacing the water from network and was kept in the alcohol before starting the drying process. The samples were placed into a drying small chamber, covered by high purity ethanol which was slowly replaced by high purity (minimum 99.8%) liquid CO_2_ with pressure 800 psi (±5%) (bone- dry LCO_2_) followed by cooling the sample to below 0 °C where they were completely covered by LCO_2_. The chamber was then slowly heated up to 40 °C at a pressure of ~1,200 psi. After heating, the chamber was cooled down to room temperature and pressure slowly dropped to ~400 psi. At that point, the chamber was opened and the dried graphene pieces were acquired. The final dried graphene foam was yielded 0.1–0.15% of the initial mass of Ni metal.

### Battery testing

The active material of graphene was weighed accurately in the range of 1 mg and it first was mixed with teflonized acetylene black (TAB-2) binder ratio (1:3 in mg) in agate mortar. The electrodes were pressed on the stainless steel collector having an area of ~2 cm^2^. The electrodes then were dried at 160 °C for 5 hours in a vacuum oven. All cells were assembled in Argon filled dry glove box. The active graphene material was used as a working electrode and Li foil as a counter electrode separated from each other by glass fiber (ADVANTEC, GB-100R) using 2032 Coin-type cell. 1 M solution of LiPF_6_ was dissolved in a 1:2 ratio (by volume) of ethylene carbonate and dimethyl carbonate, and used as an electrolyte. The galvanostatic charge-discharge measurements were carried out using the “Arbin” instrument. Discharge and charge measurements were carried out at a voltage range of 3.0–0.005 V with different current densities of 0.1–5.0 A g^−1^. The cyclic voltammetry (CV) measurements were performed at the voltage range of 3.0-0.005 V with scan speed of 1 mV s^−1^ using Bio-Logic USA Science Instruments.

### Preparation of graphene electrodes for *ex-situ* analysis

The pairs of graphene cells were assembled as described in the previous section, and discharge/charge capacity measurements were performed in 5 cycles by applying 5 mV voltage. One measurement was stopped after 5^th^ cycle when the electrode was fully discharged (Li-inserted) and another one was stopped when the electrode was charged (de-inserted). The coin cells were unsealed inside of the glove box filled with Argon gas, the electrolyte was rinsed away as much as possible using dimethyl carbonate solvent following a drying process under Argon flow. Dried graphene electrodes were sealed and packed in plastic boxes and kept in glove boxes before measurements. We referred to those electrodes or sheets as “Li-inserted” or “de-inserted” in the main text.

### Characterization of graphene foam and graphene electrodes

SEM imaging was done by SUPRA 35 VP (**ZEISS**) microscope using 5–15 KV accelerated voltage. Elemental analysis was performed using EDAX LN_2_ cooling detector. Scanning Transmission Electron Microscope (STEM) analysis (30 KV accelerated Voltage) was also performed for analyzing the inner morphology of graphene sheets.

*HR **BRUKER** D8* diffractometer with CuKα irradiation (λ = 1.54 Å) was used for XRD characterization of pristine graphene foam and graphene-based electrodes. The data were analyzed using X’Pert High Score software. The background of the patterns was subtracted automatically in a similar manner for the all curves.

XploRA Confocal Raman Microscope system (***HORIBA** Scientific)* was used for Raman characterization of all samples. Two different wavelength of excitation Laser λ = 638 nm and λ = 532 nm were applied for the characterization of pristine graphene and graphene-based electrodes. Raman mapping was performed by 5 μm (x and y axis) step under ×40 Optical lenses. LabSpec5 software was used for subtracting the background in a similar manner for each Raman spectra. The fitting of peaks and their detailed analysis was done using Origin 8.5 software.

XPS spectra were acquired using a VG Scientific MultiLab 3000 Ultra-High Vacuum (UHV) surface analysis system, equipped with a dual anode (Mg/Al) X-ray source, a VG-CLAM4 mispherical electron energy analyzer, and a 9-channel array detector. The measurements were performed at the base chamber pressure in the 10^−9^ Torr range and non-monochromatized Mg Kα X-ray (hν ≈ 1253.6 eV) was used for the excitation radiation. XPSPEAK41 analyzer software was used for interpreting the data. The shift of peaks has been calculated by interpretation of C, F and O 1 s peaks together using the data link: http://srdata.nist.gov/xps/.

A FEI Tecani F20 transmission electron microscope, operating at 200 kV and equipped with a 1024 channel Gatan Image Filter (GIF) spectrometer was used for HRTEM imaging and EELS measurements. For this study, the spectrometer was fully aligned and the full width at half maximum (FWHM) of the zero-loss peak was measured without the sample to be about 0.85 eV. High-resolution EELS spectra, acquired using an energy dispersion of 0.1 eV per channel. The carbon K-, lithium K- and oxygen K-ionization edges were collected in the diffraction mode from several areas of the sample. The pre-edge background of raw data was fitted using an exponential model and subtracted from the all data.

## Additional Information

**How to cite this article**: Paronyan, T. M. *et al*. Incommensurate Graphene Foam as a High Capacity Lithium Intercalation Anode. *Sci. Rep.* **7**, 39944; doi: 10.1038/srep39944 (2017).

**Publisher's note:** Springer Nature remains neutral with regard to jurisdictional claims in published maps and institutional affiliations.

## Supplementary Material

Supplementary Information

## References

[b1] Hérold A. Recherches sur les composés d’insertion du graphite. Bulletin de la Société chimique de France 187, 999–1012 (1955).

[b2] Wu H. & Cui Y. Designing nanostructured Si anodes for high energy lithium ion batteries. Nano Today 7, 414–429 (2012).

[b3] Zhuo P. & Zhao Y. P. A phase field model coupling lithium diffusion and stress evolution with crack propagation and application in lithium ion batteries. Physical Chemistry Chemical Physics, 17(1), 287–297 (2015). 24968175 10.1039/c4cp00563e

[b4] Zhao Y. . Self-adaptive strain-relaxation optimization for high-energy lithium storage material through crumpling of graphene. Nature Communications 5, Article number 4565, doi: 10.1038/ncomms5565 (2014). 25081187

[b5] Xiao Q. . Inward lithium-ion breathing of hierarchically porous silicon anodes. Nature Communications 6, Article number 8844, doi: 10.1038/ncomms9844 (2015). PMC4667626 26538181

[b6] Kambe N. . Intercalate ordering in first stage graphite-lithium. Mater. Sci. Eng. 40, 1–4 (1979).

[b7] Zheng T. . Lithium Insertion in High Capacity Carbonaceous Materials. J. Electrochem. Soc. 142(8), 2581–2592 (1995).

[b8] Raccichini R., Varzi A., Passerini S. & Scrosati B. The role of graphene for electrochemical energy storage. Nature Materials 14, 270–279 (2015). 10.1038/nmat4170 25532074

[b9] Dahn R., Zheng T., Liu Y. & Xue J. S. Mechanisms for Lithium Insertion in Carbonaceous Materials. Science 270, 590–593 (1995).

[b10] Sato L., Noguchi M., Demanchi A., Oki N. & Endo M. A mechanism of lithium storage in disordered carbons. Science 264, 556–558 (1994). 17732740 10.1126/science.264.5158.556

[b11] Bindra C., Nalimova V. A., Sklovsky D. E., Benes Z. & Fischer J. E. Super Dense LiC_2_ as a High Capacity Li Intercalation Anode. J. Electrochem. Soc. 145, 2377–2380 (1998).

[b12] Winter M., Besenhard J., Spahr M. E. & Novak P., Insertion Electrode Materials for Rechargeable Lithium Batteries. Advanced Materials 10, 725–763 (1998).

[b13] Dahn J. R. . Lithium Batteries: New Materials, Developments and Perspectives (G. Pistoia, Amsterdam-London-New York-Tokyo, 1994).

[b14] Zheng T., Reimers J. N. & Dahn J. R. Effect of turbostratic disorder in graphitic carbon hosts on the intercalation of lithium. Phys. Rev B 51, 734–741 (1995). 10.1103/physrevb.51.734 9978221

[b15] Pan D.. Li Storage Properties of Disordered Graphene Nanosheets. Chem. Mat. 21, 3136–3142 (2009).

[b16] D. Lian . Large reversible capacity of high quality graphene sheets as an anode material for lithium-ion batteries. Electrochimica Acta 55, 3909–3914 (2010).

[b17] Mukharajee R. . Defect-induced plating of lithium metal within porous graphene networks. Nature Communications 5, 3710(1–10) (2014). 10.1038/ncomms4710 24751669

[b18] Liu Y., Wang Y. M., Yakobson B. I. & Wood B. C. Assessing Carbon-Based Anodes for Lithium-Ion Batteries: A Universal Description of Charge-Transfer Binding Phys. Rev. Lett. 113, 028304(1–5) (2014). 10.1103/PhysRevLett.113.028304 25062244

[b19] Datta D., Li J., Koratkar N. & Shenoy V. B. Enhanced lithiation in defective graphene. Carbon 80, 305–310 (2014).

[b20] McCann E. Asymmetry gap in the electronic band structure of bilayer graphene. Phys. Rev. B 74, 161403(1–4) (2006).

[b21] Lopes dos Santos J. M. B., Peres N. M. R. & Castro Neto A. H. Graphene Bilayer with a Twist: Electronic Structure. Phy. Rev. Lett. 99, 256802(1–4) (2007). 10.1103/PhysRevLett.99.256802 18233543

[b22] Li G. . Observation of Van Hove singularities in twisted graphene layers. Nature Physics 6, 109–113 (2010).

[b23] Latil S., Menuier V. & Henrard L. Massless fermions in multilayer graphitic systems with misoriented layers: *Ab initio* calculations and experimental fingerprints. Phys. Rev. B 76, 201402(1–4) (2007).

[b24] Berashevich J. & Chakraborty T. Interlayer repulsion and decoupling effects in stacked turbostratic graphene flakes. Phys. Rev. B 84, 033403(1–4) (2011).

[b25] Wood B. V., Ogitsu T., Otani M. & Biener J. First-Principles-Inspired Design Strategies for Graphene-Based Supercapacitor Electrodes. J. Phys. Chem. C 118, 4–15 (2014).

[b26] Li X. . Large-Area Synthesis of High-quality and uniform graphene films on Copper foils. Science 324, 1312–1314 (2009). 19423775 10.1126/science.1171245

[b27] Yu Q. . Graphene segregated on Ni surfaces and transferred to insulators. Appl. Phys. Lett. 93, 113103(1–3) (2008).

[b28] Paronyan T. M., Pigos E. M., Chen G. & Harutyunyan A. R. The Formation of Ripples in Graphene as a Result of Interfacial Instabilities, ACS NANO 5(12), pp. 9619–9627 (2011). 22092098 10.1021/nn202972f

[b29] Chen Z. . Three-dimensional flexible and conductive interconnected graphene networks grown by chemical vapour deposition. Nature Materials 10, 424–428 (2011). 21478883 10.1038/nmat3001

[b30] Zhou S., Xu J., Xiao Y., Zhao N. & Wong C.-P. Low-temperature Ni particle-templated chemical vapor deposition growth of curved graphene for supercapacitor applications. Nano Energy 13, 458–466 (2015).

[b31] Paronyan T. M. & Harutyunyan A. R. Graphene originated 3D structures grown on the assembled nickel particles. APS March Meeting 58, B B6.00005 (2013).

[b32] Warner H., Rümmeli M. H., Gemming T., Bücher B. & Broggs G. A. D. Direct imaging of rotational stacking faults in few layer graphene, Nano Letters 9, 102–106 (2009). 19072722 10.1021/nl8025949

[b33] Ferrari A. C. & Basko D. M. Raman spectroscopy as a versatile tool for studying the properties of graphene. Nature Nanotechnology 8, 235–246 (2013). 10.1038/nnano.2013.46 23552117

[b34] Malard L. M., Pimenta M. A., Dresselhaus G. & Dresselhaus M. S. Raman spectroscopy in graphene. Physics Reports 473, 51–87 (2009).

[b35] Ponchara P., Ayari A., Michel T. & Sauvajol J.-L. Raman spectra of misoriented bilayer graphene. Phys. Rev. B 78, 113407(1–4) (2008).

[b36] Lui C. H. . Imaging Stacking Order in Few-Layer Graphene. Nano Lett. 11, 164–169 (2011). 21121668 10.1021/nl1032827

[b37] Kim K. . Raman Spectroscopy Study of Rotated Double-Layer Graphene: Misorientation-Angle Dependence of Electronic Structure. Phys. Rev. Let. 108, 246103(1–6) (2012). 10.1103/PhysRevLett.108.246103 23004295

[b38] Wu J.-B. . Resonant Raman spectroscopy of twisted multilayer graphene. Nature Communications 5, 5309(1–10) (2014). 10.1038/ncomms6309 25382099

[b39] Tuinstra F. & Koenig J. L. Raman Spectrum of Graphite. J. Phys. Chem. 53, 1126–1130 (1970).

[b40] Cançado L. G. . General equation for the determination of the crystallite size *L_a_* of nanographite by Raman spectroscopy. Applied Physics Letters 88, 163106(1)–(3) (2006).

[b41] Eckmann A. . Probing the Nature of Defects in Graphene by Raman Spectroscopy. Nano Lett. 12, 3925 (2012). 22764888 10.1021/nl300901a

[b42] Boehm R. C. & Bannerjee A. Theoretical study of lithium intercalated graphite. J. Chem. Phys. 96, 1150–1157 (1992).

[b43] Liu M. Y., Xue J. S., Zheng T. & Dahn J. R. Mechanism of lithium insertion in hard carbons prepared by pyrolysis of epoxy resins. Carbon 34, 193–200 (1996).

[b44] Rytkönen K., Akola J. & Manninen M. Density functional study of alkali-metal atoms and monolayers on graphite (0001). Phys. Rev. B 75, 075401(1–9) (2007).

[b45] Inaba M. . *In Situ* Raman Study on Electrochemical Li Intercalation into Graphite. J. Electrochem. Soc. 142(1), 20–26 (1995).

[b46] Pollak E. . The Interaction of Li+with Single-Layer and Few-Layer Graphene. Nano Letters 10, 3386–3388 (2010). 20677788 10.1021/nl101223k

[b47] Sato K. . Raman spectra of out-of-plane phonons in bilayer graphene. Physical Review B 84, 035419(1–5) (2011).

[b48] Sole C., Drewett N. E. & Hardwick L. J. *In situ* Raman study of lithium-ion intercalation into microcrystalline graphite. Faraday Discussions 172, 223–237 (2014). 25427224 10.1039/c4fd00079j

[b49] Venezuela P., Lazzeri M. & Mauri F. Theory of double-resonant Raman spectra in graphene: Intensity and line shape of defect-induced and two-phonon bands. Phys Rev B 84, 035433 (2011).

[b50] Mordkovich V. Z. Synthesis and XPS investigation of superdense lithium-graphite intercalation compound, LiC2. Synthetic materials 80, 243–247 (1996).

[b51] Wang F. . Chemical Distribution and Bonding of Lithium in Intercalated Graphite: Identification with Optimized Electron Energy Loss Spectroscopy. ASC NANO 5, 1190–1197 (2011). 10.1021/nn1028168 21218844

