# Supplementary material for: Incommensurate Graphene Foam as a High Capacity Lithium Intercalation Anode
Source: Sci Rep. 2017 Jan 6;7:39944. doi: 10.1038/srep39944 (PMC5216342; doi:10.1038/srep39944)
Supplement: Supplementary Information [file srep39944-s1.pdf]

Supplementary Materials for

# Incommensurate Graphene Foam as a High Capacity Lithium Intercalation Anode

Tereza M. Paronyan*[1], Arjun Kumar Thapa[2], Andriy Sherehiy[3], Jacek B. Jasinski[2], John Samuel Dilip Jangam[2,4]

[1] Speed School of Engineering, University of Louisville, 2210 S. Brook st., Louisville, KY, 40208, USA

Correspondence and requests for materials should be addressed to Tereza Paronyan email addresses; tereza.paronyan@louisville.edu, teparonyan@gmail.com

[2] Conn Center of Renewable Energy Research, University of Louisville, KY, USA

[3] ElectroOptics Research Institute and Nanotechnology Center, University of Louisville, KY, USA

[4] Department of Industrial Engineering, University of Louisville, KY, USA

## Supplementary Text

**Estimation of crystallite size and density of defects**

The intensity ratio of D and G bands can determine the crystallite size ($L_{a)}$ of graphene in plane by the equation proposed in Ref. 40

$$L_a = (2.4 \times 10^{-10})\lambda^4_{laser(nm)}\left(\frac{I_D}{I_G}\right)^{-1} \qquad (1),$$

where $\lambda_{laser}$ = 638 nm is the excitation laser, $I_D$ and $I_G$ are the intensities of D and G bands, respectively.

$\frac{I_D}{I_G} = 0.07 - 0.085$

$L_a$= $(2.4 \times 10^{-10})638^4 \times 1/0.07 = 568\,nm$ for $\frac{I_D}{I_G} = 0.07$ and

$L_a$ = 467.8 nm for $\frac{I_D}{I_G} = 0.085$

The concentration of defects $N_D$ in the graphene is calculated using the formula (6) proposed in Ref. 40.

$$N_D(cm^{-2}) = \frac{(1.8 \pm 0.5)\cdot 10^{22}}{\lambda^4_{Laser(nm)}}\frac{I_D}{I_G} \approx 8 \times 10^9 \qquad (2),$$

where $I_D$ and $I_G$ are the heights of D and G bands, respectively,

$\lambda_{laser}$ = 638 nm is the wavelength of excitation laser.

The estimated carbon concentration in graphene $N_C = 3.9 \times 10^{15}$ cm$^{-2}$, thus ratio of $N_D/N_c = 2.05 \times 10^{-6}$ was obtained extremely low equivalent ~0.02% of carbon.

**Estimation of incommensurate percentage by Raman analysis**

To estimate the degree of incommensurateness of our graphene foam samples we evaluated several large area (100-200 µm by x and y axes) Raman mapping for each sample and analyzed hundreds of individual spectra by Lorentzian fit of G and 2D bands.

We collected the range of 2D peak bandwidth (referred as FWHM) and $I_{2D}/I_G$ values when single Lorentzian is in better fit than multi-Lorentzian using Origin 8.5 software. The FWHM of 2D peak and $I_{2D}/I_G$ values for 45 most common individual spectra to find the boundary of those two values between single-(*green dots*) and multi-Lorentzian (*blue dots*) fits of 2D band (Table S1). To gather these two values for hundreds of spectra, we generate fit of G and 2D peaks with a single Lorentzian peak for simplicity for all the spectra. The fit was chosen based on the adjusted R-square value of the coverage summary curve of Lorentzian components. We found the lowest value of FWHM of 2D of our samples is 28 $cm^{-1}$ ($I_{2D}/I_G$ = 2.63) with single Lorentzian fit which agrees to the experimentally measured values of single-layer graphene (SLG). We consider commensurate (Bernal) bilayer graphene would have at least 56 $cm^{-1}$ (double of SLG) of FWHM of 2D. Thus, we choose FWHM = 56 $cm^{-1}$ of 2D as an upper boundary of incommensurate multilayer graphene when $I_{2D}/I_G$ varies freely and that is in good agreement of our analyzed data (table S1) with single- Lorentzian fit. On the other hand, 2D peak can be broadened due to rotation angles between layers if graphene is an incommensurate state. We found FWHM = 56-65 $cm^{-1}$ when $I_{2D}/I_G \geq 0.94$, the single-Lorentzian of 2D is still in good fit. Thus, we estimate incommensurateness degree of bulk foam by analyzing the hundreds of spectra (batch processing in Origin 8.5) based on the set of these two values (Table S2).

**<u>Calculation of specific capacity for graphene having a finite number of layers</u>**

The specific capacity of graphite $LiC_6$ is known to be 372 mAh $g^{-1}$. We determined the Sp.C. for other possible stoichiometry following the same logic considering each hexagonal ring can host one lithium atom. We consider the ideal case

with a single layer graphene, and Li intercalates on both sides of the graphene plane to each hexagonal ring of carbon (Fig. 6F). This is most easily seen starting from a single layer of graphene with lithium atoms above and below each hexagon. Each of the 6 carbon atoms (each hexagon) is shared between three hexagons, so that the stoichiometry is 2 lithium atoms per (1/3) × 6 =2 carbon atoms, giving $Li_2C_2$.This is 6 times more lithium storage than $LiC_6$ which then gives a capacity of 2,232 mAh $g^{-1}$. However, for purposes of calculation, the N=1 estimate is easily extended for additional layers.

We calculate theoretical specific capacity $C_{s,th.}$ using the following general formula (3) from ref. Supplementary 1

$$C_{s,th} = \frac{nF}{M} \quad (3)$$

where, $n$ is the number of moles of electrons transferred per mole;

$F$ – Faraday's constant,

$F = 9.6485 \cdot \frac{10^4 \mathrm{C}}{\mathrm{mole\ \bar{e}}}$; $M$ is the molar mass of active material.

In our case, the equation of specific capacity $C_{s,th,N}$ would be expressed in terms of number of graphene layers $N$ by equation (4). If we consider ($N$+1) valence electrons (atoms) of Li per molar mass of 1/3 carbon, then for each hexagon it would be 1/3×6×12.01×$N$ (per 1/3 carbon for 6$N$ carbon atoms) depending on the number of carbon layers $N$.

$$C_{s,th,N} = \left(\frac{N+1}{N} \cdot \frac{1 mole\ \bar{e}}{12.01 \cdot 6 \cdot \frac{1}{3}\ [g]}\right) \cdot \left(9.6485 \cdot 10^4 \left[\frac{Coulomb}{mole\ \bar{e}}\right]\right) \cdot 1 \cdot \left[\frac{Amp \cdot sec}{Coulomb}\right] \cdot \left[\frac{1\ hour}{3{,}600\ sec}\right] = 1{,}116 \cdot \left(\frac{N+1}{N}\right) \left[\frac{mAh}{g}\right] = 2{,}232 \cdot \left(\frac{N+1}{2N}\right) \left[\frac{mAh}{g}\right] \quad (4)$$

where, $N$ is the number of graphene layers,

$9.6485 \times \frac{10^4 C}{mole\ \bar{e}}$ - is the Faraday constant.

N = 2, $Sp.C. = 2{,}232 \cdot \left(\frac{2+1}{2 \cdot 2}\right) \left[\frac{mAh}{g}\right] = 1{,}674 \cdot \left[\frac{mA \times h}{g}\right]$

$N$ = 3, $Sp.C. = 2{,}232 \cdot \left(\frac{3+1}{2 \cdot 3}\right) \left[\frac{mAh}{g}\right] = \frac{4}{6} \cdot 2{,}232 \left[\frac{mAh}{g}\right] = 1{,}488 \left[\frac{mAh}{g}\right]$

For a bilayer graphene (N=2), one layer each of graphene and of lithium is added to the single layer graphene. This increases the number of lithium atoms per stack of hexagons by one and doubles the number of carbon atoms, which corresponds to 3 lithium atoms for each 4 carbon atoms, giving $Li_3C_4$. Continuing this process of adding a layer of graphene and lithium gives the overall stoichiometry of $Li_{N+1}C_{2N}$ where N is the number of graphene layers in the stack. Thus an infinite stack would approach to the stoichiometry of $LiC_2$ (capacity of 1,116 mAh $g^{-1}$). The capacity values for few layers presented in Table S4.

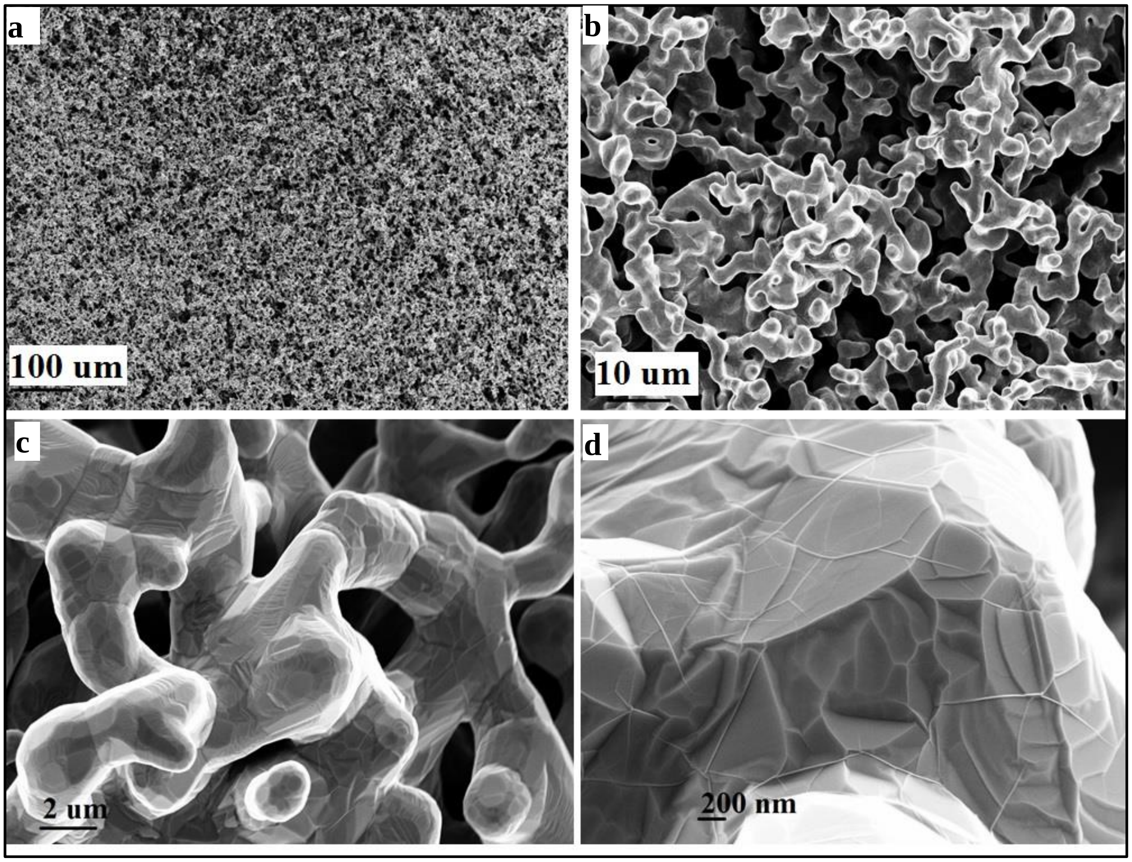


**Supplementary Fig. 1**. **SEM images of graphene film on Ni catalyst particles.** (**a-d**) Images at various magnifications. The growth was processed at 1,025°C using $CH_4$ ~8sccm rate with $Ar:H_2$ (3:2) carrier gas ~80 sccm flow rate.

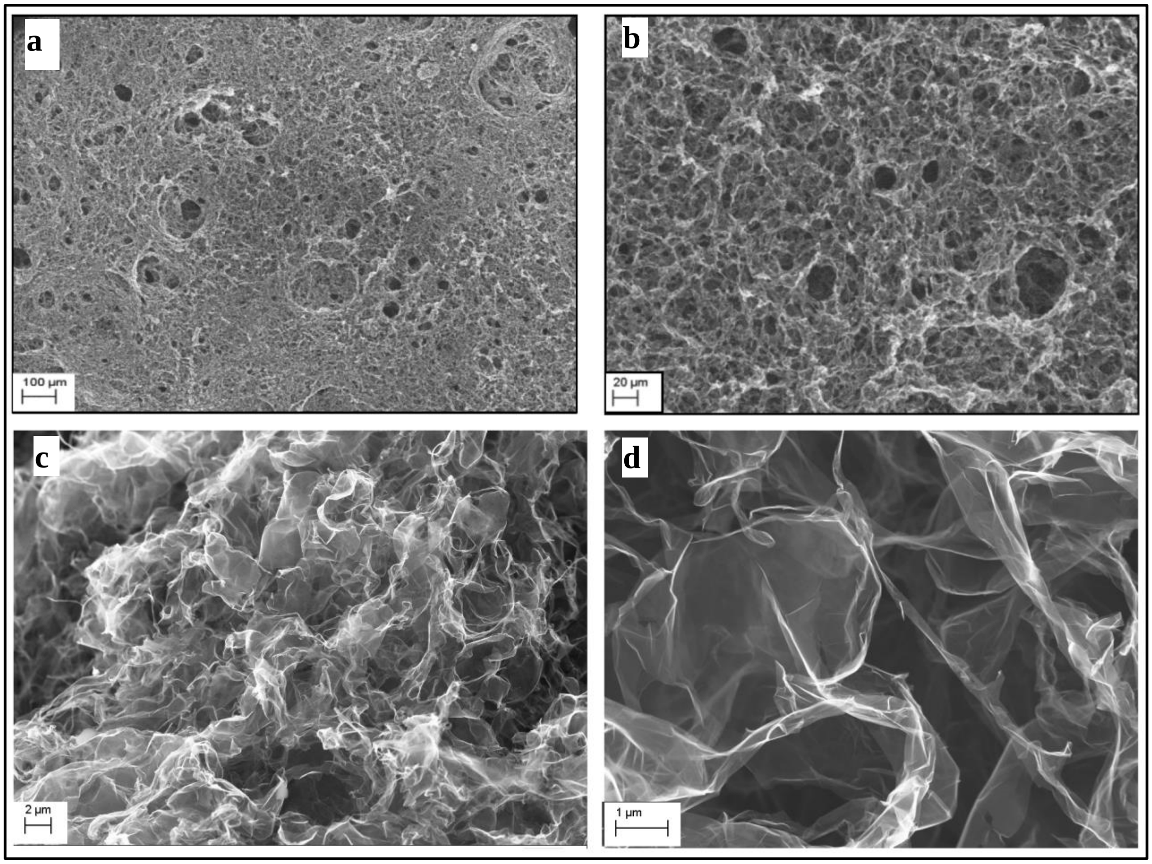


**Supplementary Fig. 2**. **SEM images of graphene foam**. **(a)-(d)**The images of the nickel-free graphene foam at various magnifications.

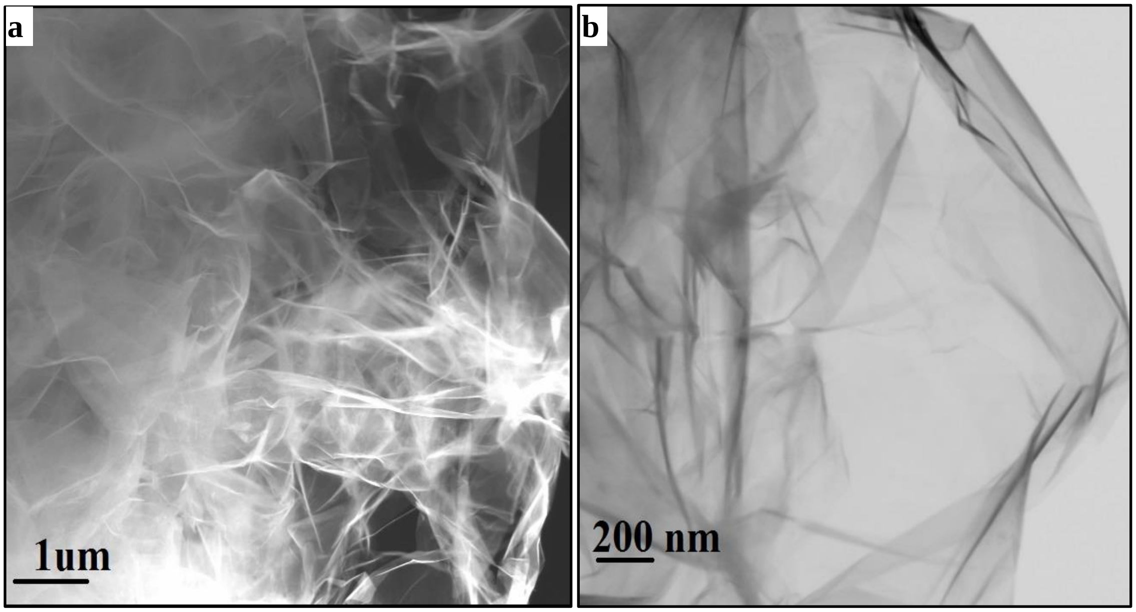


**Supplementary Fig. 3. Scanning Transmission Electron Microscope (STEM) images of graphene sheet**.

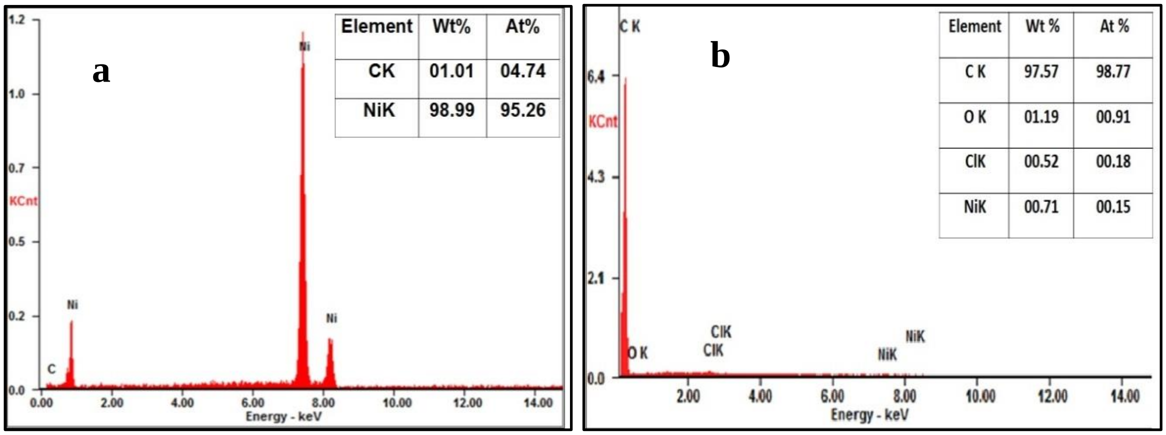


**Supplementary Fig. 4. EDS analysis of graphene network**. The insets show elemental analysis. **(a)** EDS spectra of graphene film with Nickel template (growth at 1,025°C using $CH_4$ ~8 sccm rate with Ar : $H_2$ (3:2) carrier gas ~80 sccm flowing rate). **(b)** EDS spectra of pristine graphene network after etching the Nickel followed to rinse in DI-water and dry by CPD.

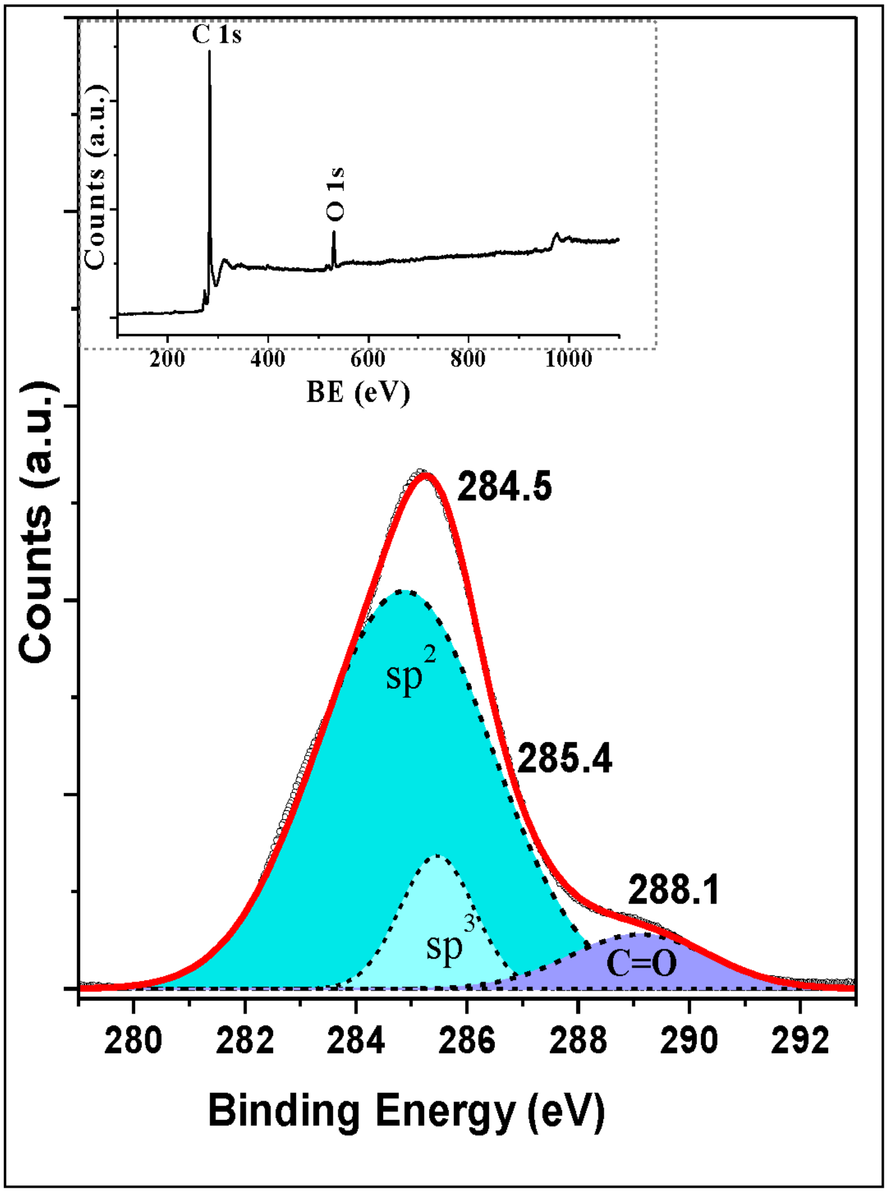


**Supplementary Fig. 5. XPS spectra of C 1s of graphene foam.** The inset shows the full XPS spectrum (survey).

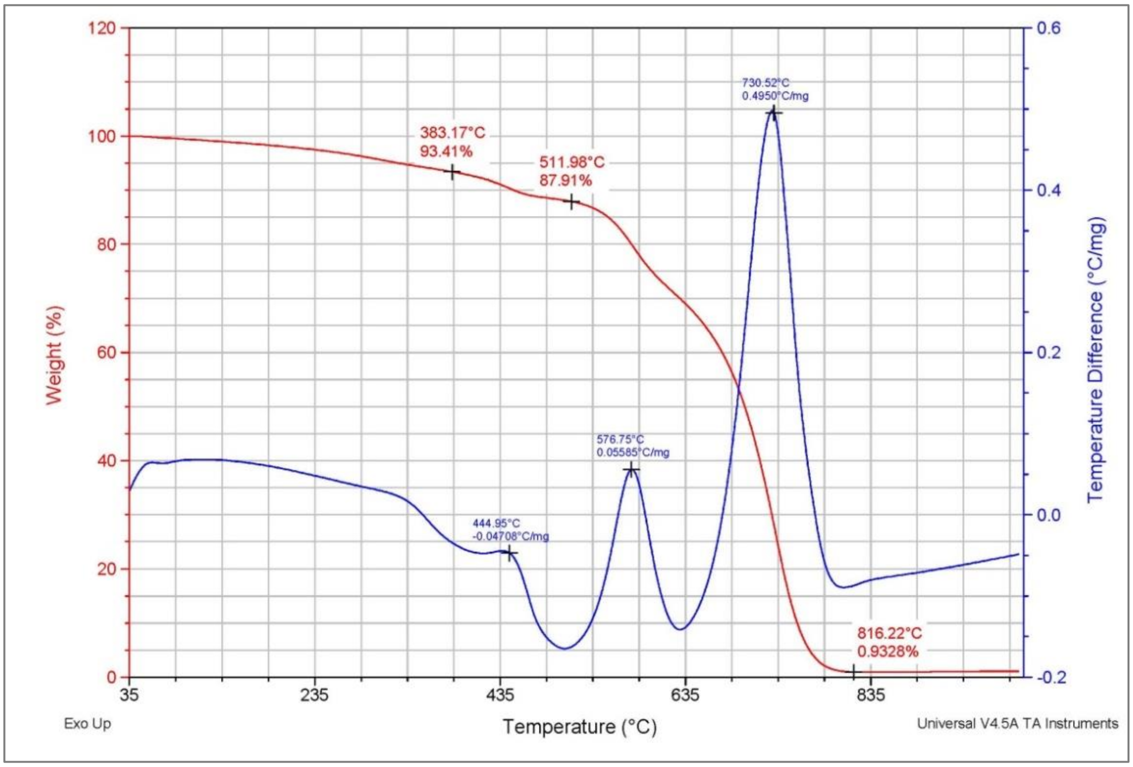


**Supplementary Fig. 6. Thermogravimetric analysis of graphene foam**. Dry graphene foam was heated by 1C°/min rate under air flow. The weight losses at 383 °C is associated to the amorphous carbon. The residual 0.93% at 816°C is the Nickel nanoparticles as it checked by SEM/EDS.

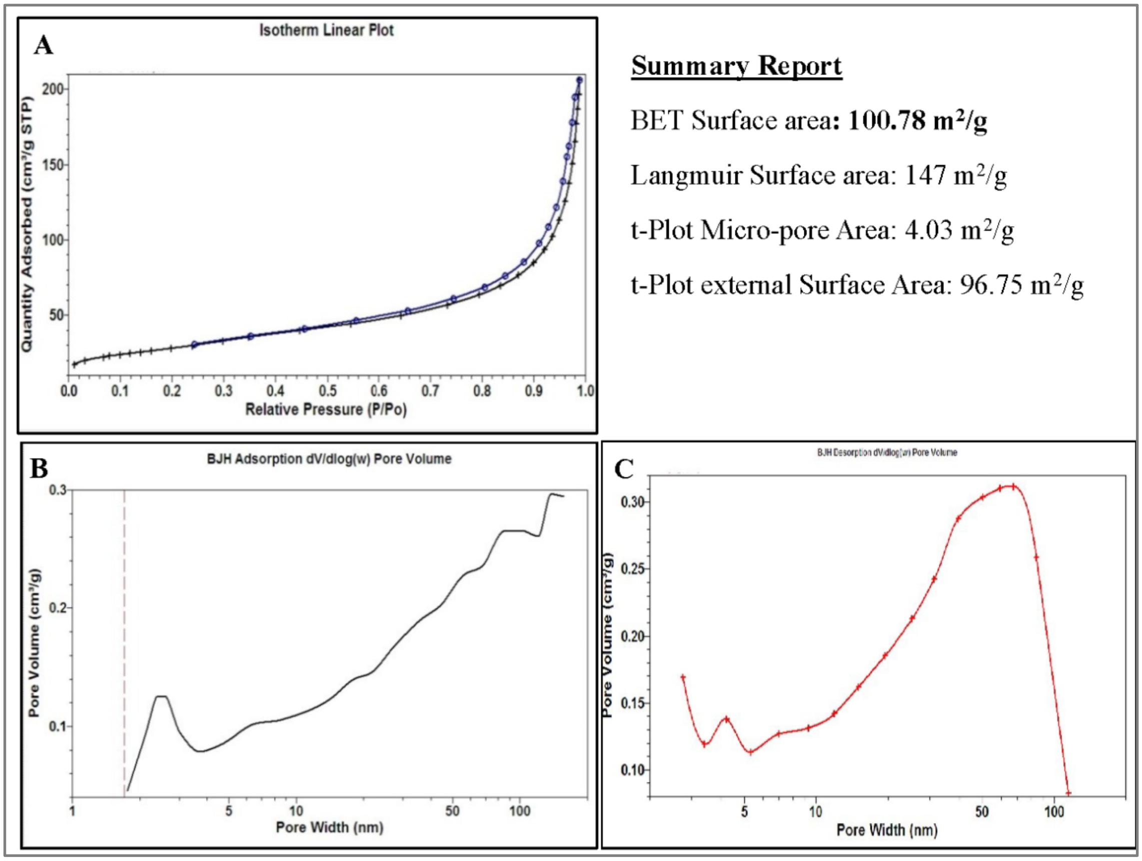


**Supplementary Fig. 7. BET results of IMLG graphene foam with 93% incommensurateness (Sample 1)**. The Specific Surface Area (SSA) was measured based on nitrogen gas absorption. (**a**) $N_2$ molecules adsorption/desorption isotherms. (**b**) $N_2$ molecules adsorption and (**c**) desorption curve for per volume.

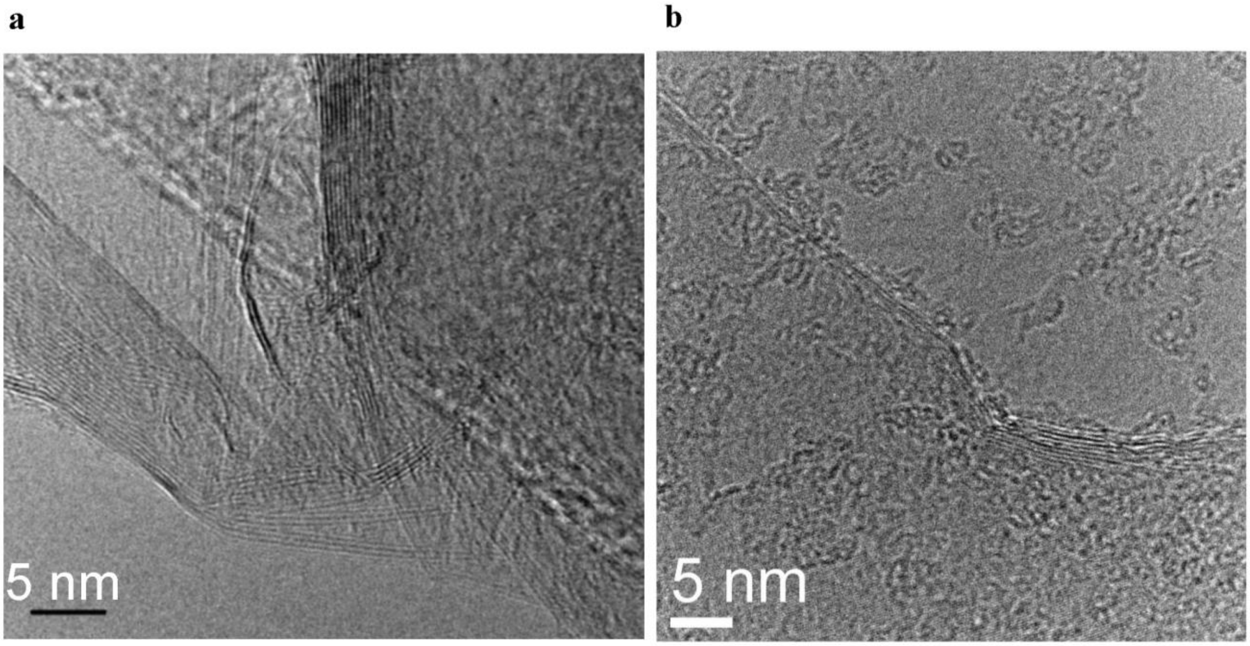

**Supplementary Fig. 8. HRTEM images of incommensurate multilayer sheets (a, b).**

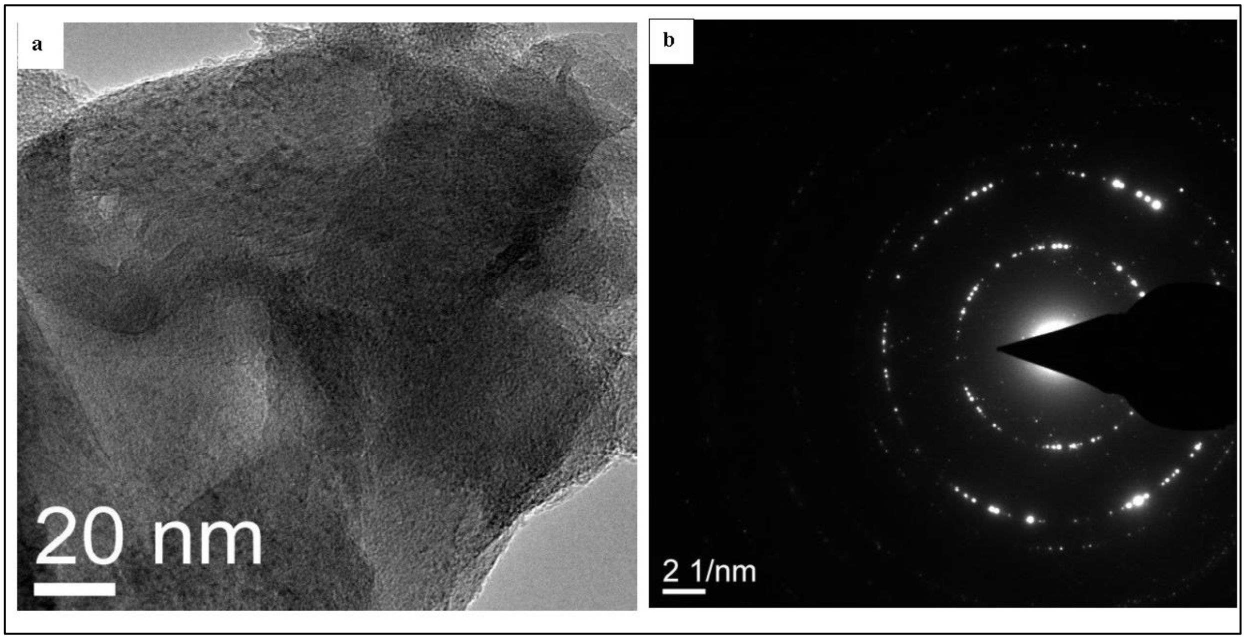


**Supplementary Fig. 9. SAED analysis of pristine graphene sheets**. (**a**) HRTEM image of incommensurate multilayer sheets. (**b**) SAED pattern of the same area.

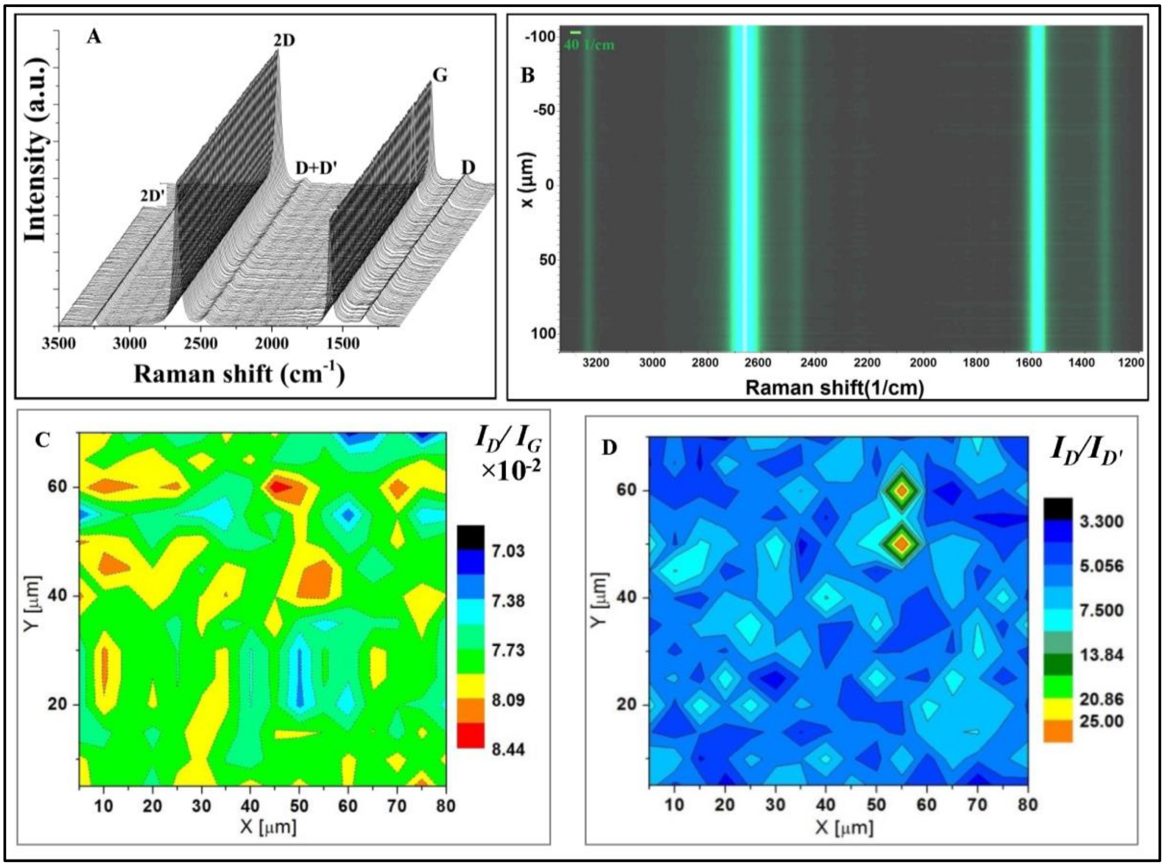


**Supplementary Fig. 10. Raman mapping analysis of pristine graphene foam** performed by 5×5 (X,Y) μm step (λ = 638 nm laser wavelength). (**a**) 3D plot of 238 spectra of single mapping area. (**b**) 2D colored image for the same area evaluated by Raman intensity. (**c**) Mapping analysis of $I_D/I_G$ of the same area. (**d**) Mapping analysis of $I_D/I_{D'}$ of the same area.

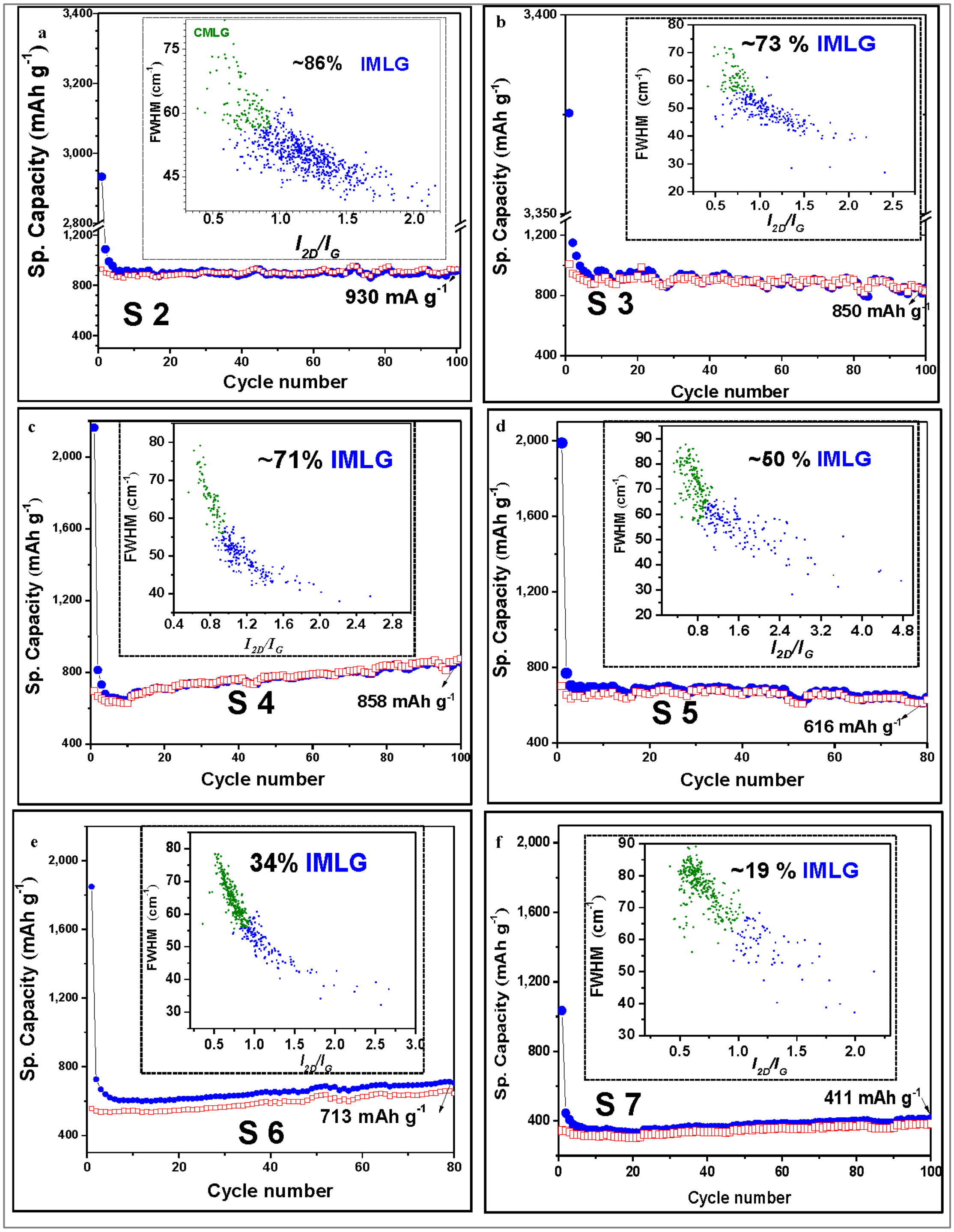


**Supplementary Fig. 11. Charge-discharge cycling of Samples 2-7 at 100 mA g$^{-1}$ current density, (a)-(f).** The insets show scattergrams of FWHM of 2D peak vs $I_{2D}/I_G$ by Raman mapping analysis for each pristine samples used for battery test.

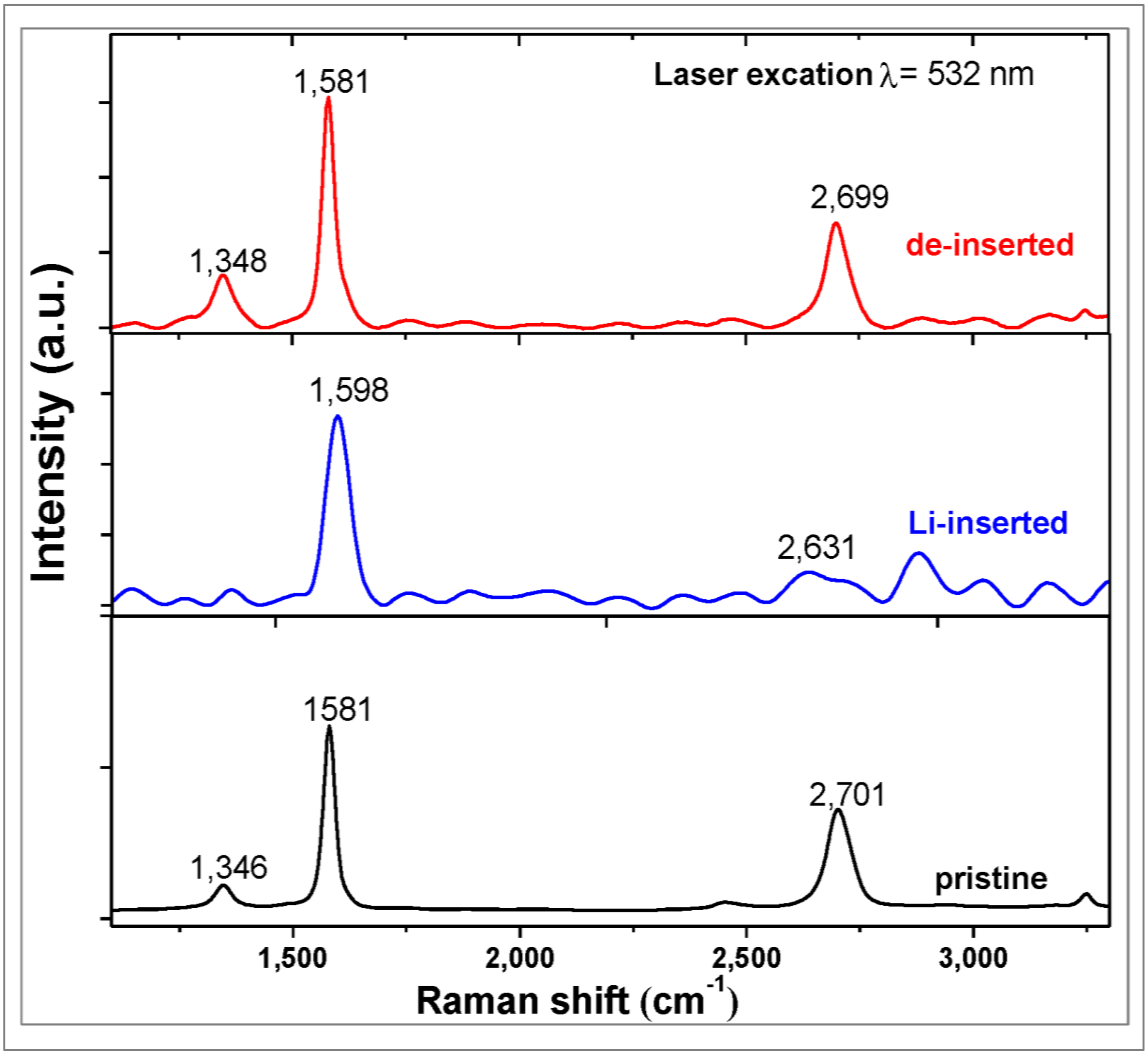


**Supplementary Fig. 12. Raman spectra of IMLG-based electrodes with Laser wavelength λ = 532 nm** proceed by 5×5 µm (X,Y) mapping step. (*blue curve)* - Li-inserted electrodes. (*red curve)* de-inserted electrodes. (*black curve)* -averaged Raman spectrum of 125 individual spectra of pristine IMLG foam (Sample 1).

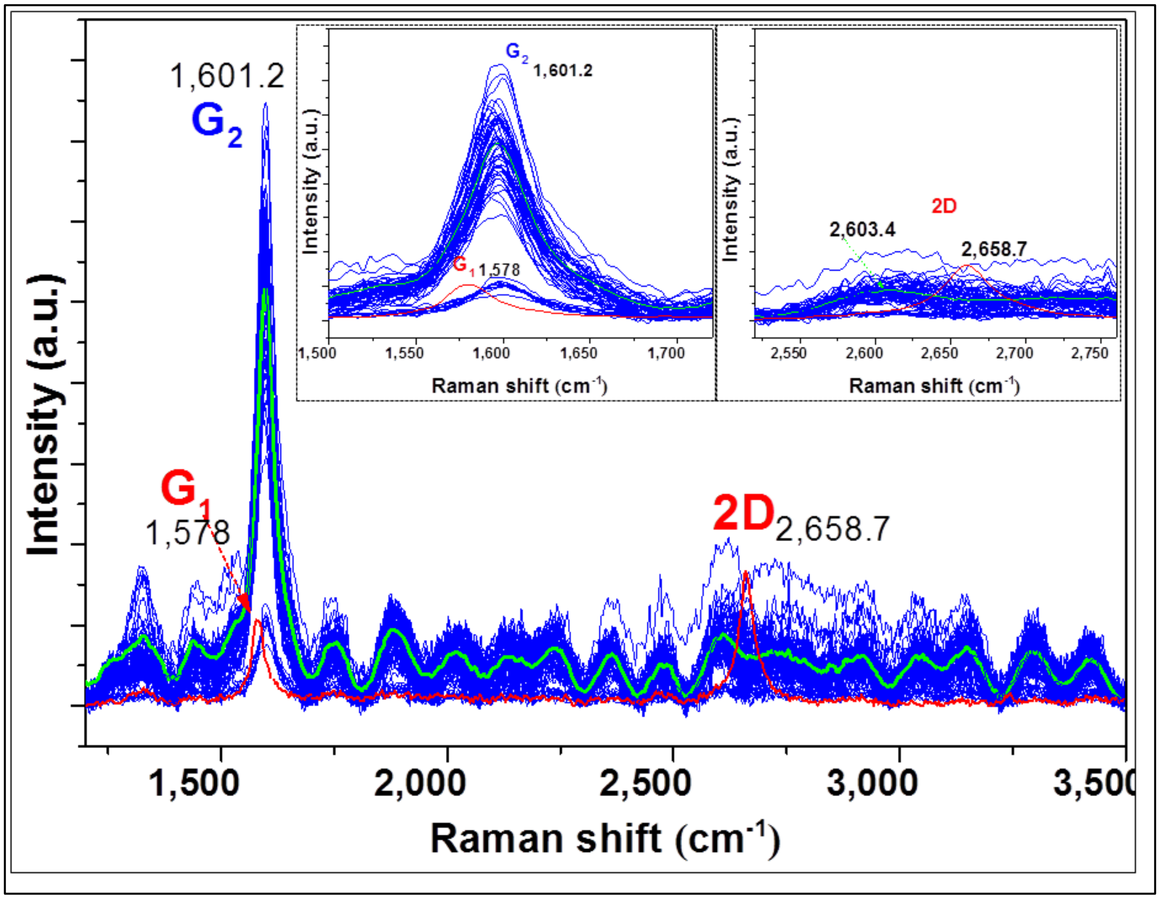


**Supplementary Fig. 13.** ***Ex-situ*** **Raman mapping spectra of Li-inserted IMLG-based electrodes (unexposed) after 5th cycle** (λ = 638 nm laser wavelength)**.** *(red* curve)- the individual spectrum without intercalated Li. (*green* curve)- the averaged spectrum of all 80 individual spectra presented in *blue*. The insets show G (*left*) and 2D peaks expended range (*right*).

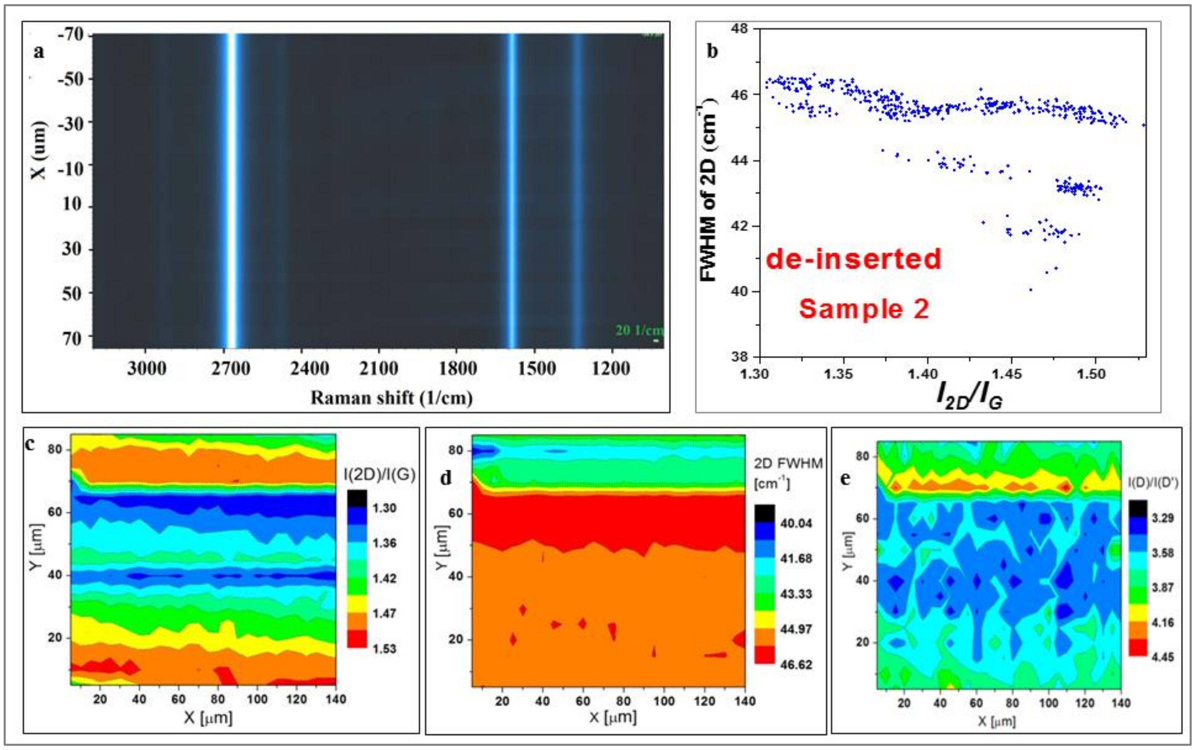


**Supplementary Fig. 14. Raman analysis of de-inserted graphene electrodes after 100th cycle** (λ = 638 nm laser wavelength) performed by 5×5 (X,Y) µm mapping step for 488 spectra. (**a**) 2D colored image of map by Raman intensity. **(b)** Scattergram of FWHM of 2D vs. $I_{2D}/I_G$ of all 488 spectra. Mapping analysis of $I_{2D}/I_G$ (**c**) and **(d)** FWHM of 2D band and $I_D/I_{D'}$.

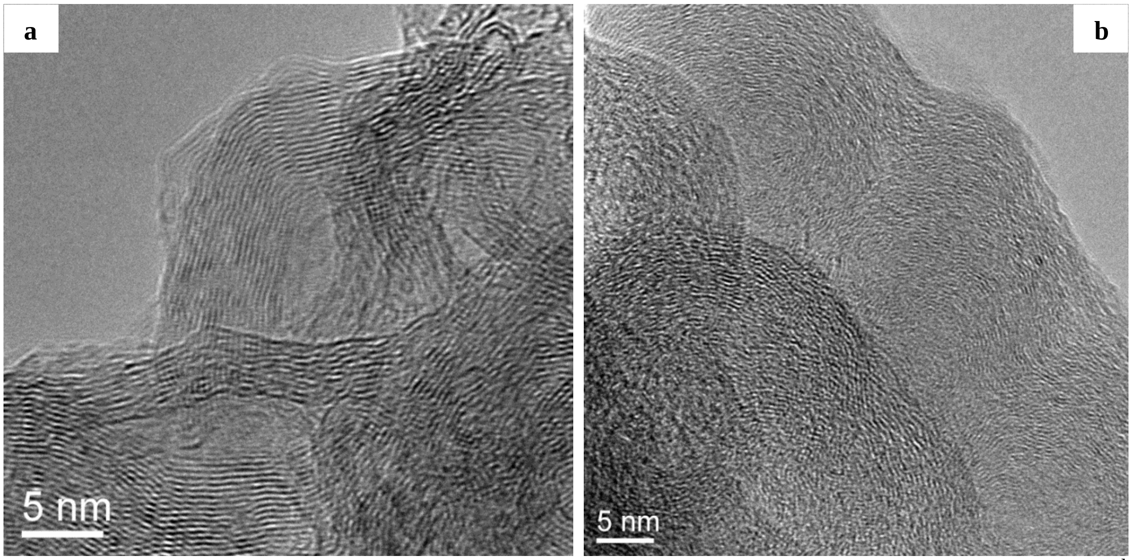


**Supplementary Fig. 15. HRTEM images of de-inserted graphene sheets. (a**)- after 5th cycle**, (b)-** after 100th cycle.

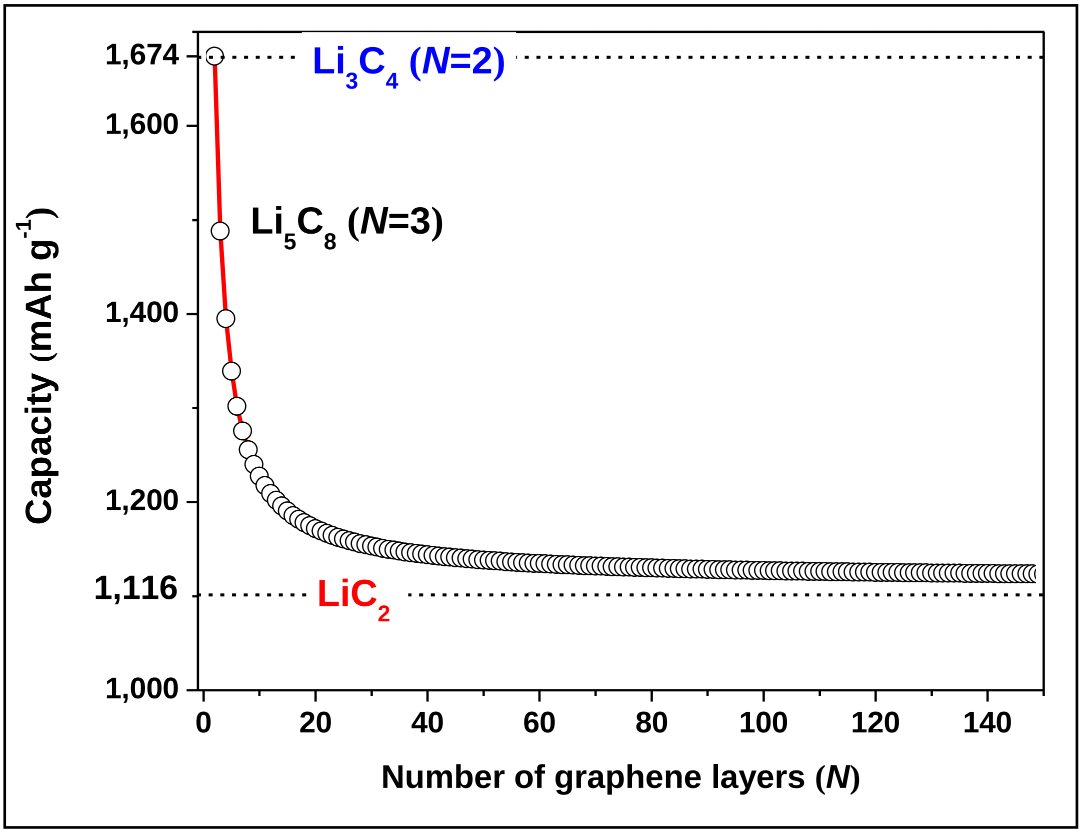


**Supplementary Fig. 16.** The dependency of theoretical capacity values versus on graphene layers based on $Li_{N+1}C_{2N}$ stoichiometric formula.

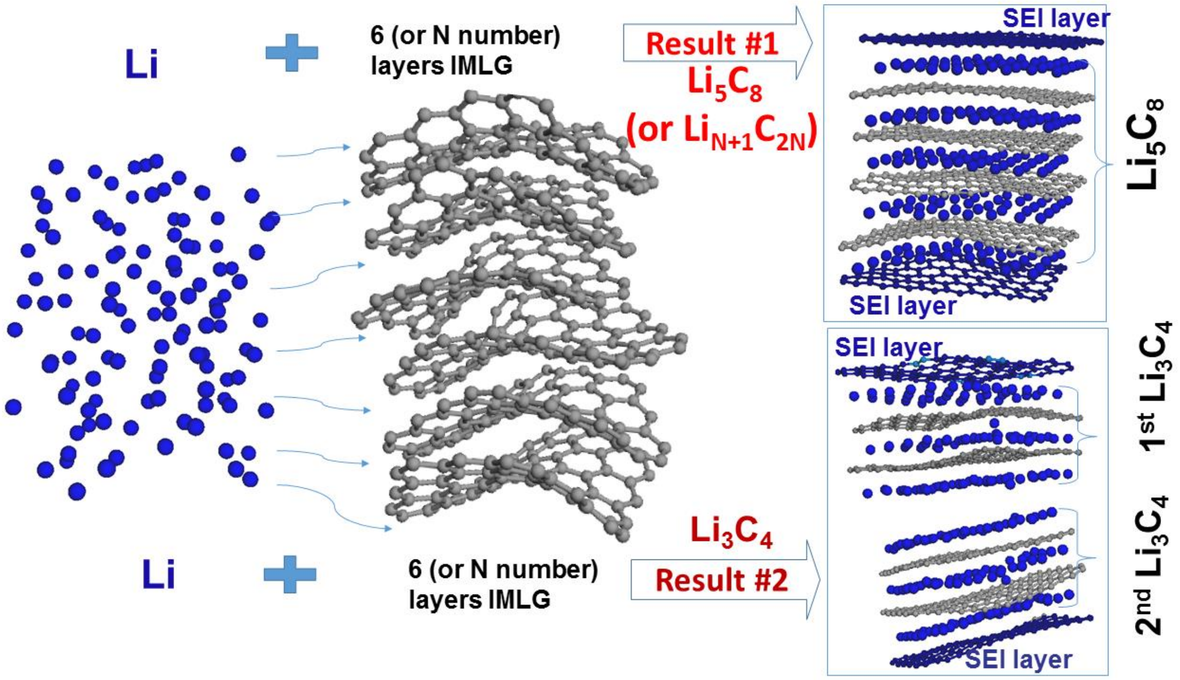


**Supplementary Fig. 17.** Schematic illustration of lithium intercalation into six (or N number) layers IMLG recombining either multilayer (result #1) or bilayer (result #2) at the second cycle.

**Supplementary Table 1. The combination of FWHM (2D band) and $I_{2D}/I_G$ *values* for 45 individual spectra**. The fit for each 2D peak was performed both single and multi-Lorentzian. The FWHM values are defined by simplified single- Lorentzian fit.

| Spectra # | $I_{2D}/I_G$ | FWHM of 2D band ($cm^{-1}$) | |
|---|---|---|---|
| | | Single- Lorentzian fit (IMLG) | Multi-Lorentzian fit (CMLG) |
| 1 | 2.26 | 45.8 | |
| 2 | 4.8 | 33.49 | |
| 3 | 0.94 | 60.33 | |
| 4 | 0.54 | | 81.4 |
| 5 | 1.09 | 58.53 | |
| 6 | 1.45 | 46.63 | |
| 7 | 0.62 | | 86.2 |
| 8 | 0.82 | | 66.45 |
| 9 | 1.06 | 62.7 | |
| 10 | 1.17 | 57.49 | |
| 11 | 0.58 | | 80.2 |
| 12 | 0.68 | | 81.7 |
| 13 | 1.73 | 48.45 | |
| 14 | 1.82 | 46.79 | |
| 15 | 2.95 | 31.13 | |
| 16 | 1.07 | 57.12 | |
| 17 | 1.03 | 60.69 | |
| 18 | 1.25 | 58.21 | |
| 19 | 1.19 | 54.2 | |
| 20 | 2.49 | 36.2 | |
| 21 | 0.95 | 62.5 | |
| 22 | 1.33 | 50.84 | |
| 23 | 0.63 | | 77.7 |
| 24 | 1.09 | 56.3 | |
| 25 | 0.81 | | 67.1 |
| 26 | 0.67 | | 63 |
| 27 | 1.19 | 53.6 | |
| 28 | 0.8 | 49.6 | |
| 29 | 0.8 | 52.3 | |
| 30 | 0.74 | | 64.7 |

| | | | |
|---|---|---|---|
| 31 | 0.96 | | 69.66 |
| 32 | 2.63 | 28 | |
| 33 | 3.39 | 43 | |
| 34 | 0.98 | 59.67 | |
| 35 | 0.89 | 57.7 | |
| 36 | 0.92 | | 64.91 |
| 37 | 0.82 | 50.3 | |
| 38 | 0.8 | | 71.5 |
| 39 | 4.37 | 37 | |
| 40 | 3.5 | 39.2 | |
| 41 | 4.37 | 37 | |
| 42 | 0.62 | | 86 |
| 43 | 2.61 | 39.9 | |
| 44 | 4.81 | 33.48 | |
| 45 | 4.02 | 37.5 | |

***Supplementary table 2.*** **The set of FWHM and** *$I_{2D}/I_G$* **to classify** ***commensurate*** **or** ***incommensurate*** **boundaries** presented in Fig. 2f and Supplementary Fig. 11**.**

| ***Stacking order*** | ***FWHM (cm$^{-1}$)*** **↔** | ***$I_{2D}/I_G$*** |
|---|---|---|
| Incommensurate | ≤ 56 ↔ | ~ |
| Incommensurate | 56-65 ↔ | ≥ 0.94 |
| Commensurate or mixture | > 56 ↔ | 0.3 -0.94 |

**Supplementary table 3. EC measurements data of Samples 1-7 and graphite** presented in Fig. 3**.**

| **Sample #** | Incommensurate degree of pristine graphene, % | **Discharge Capacity,** mAh $g^{-1}$ | | | **Coulombic efficiency** at $80^{th}$ or $100^{th}$ cycle, % |
|---|---|---|---|---|---|
| | | **$1^{st}$ cycle** | **$2^{nd}$ cycle** | **$80^{th}$ or $100^{th}$ cycle** | |
| Sample 1 (S2) | 93 | 3,302 | 1,542 | **1,539** | **75** |
| Sample 2 (S1) | 86 | 2,933 | 1,087 | **928** | **100** |
| Sample 3 (S3) | 76 | 3,375 | 1,149 | 858 | 98 |
| Sample 4 (S4) | 73 | 2,162 | 813 | 850 | 102 |
| Sample 5 (S5) | 50 | 1,987 | 768.8 | 616* | 99* |
| Sample 6 (S6) | 34 | 1,848 | 726 | 713* | 92* |
| Sample 7 (S7) | 19 | 1,033 | 444 | 411 | 94 |
| *graphite* | *2* | *365* | *325* | *250* | *99* |
| *- cells were run only 80 cycles. | | | | | |

**Supplementary table 4. Calculated capacities for 2-10 layers of graphene.**

| **Number of layers (N)** | **Stoichiometric formula $Li_{N+1}C_{2N}$** | **Capacity (mAh $g^{-1}$)** |
|---|---|---|
| 2 | $Li_3C_4$ | 1,674 |
| 3 | $Li_4C_6$ | 1,488 |
| 4 | $Li_5C_8$ | 1,395 |
| 5 | $Li_6C_{10}$ | 1,339 |
| 6 | $Li_7C_{12}$ | 1,302 |
| 7 | $Li_8C_{14}$ | 1,275 |
| 8 | $Li_9C_{16}$ | 1,256 |
| 9 | $Li_{10}C_{18}$ | 1,240 |
| 10 | $Li_{11}C_{20}$ | 1,228 |

## References

**Supplementary 1**. Glaize, C., Genies, S., *Lead-Nickel Electrochemical Batteries* (ISTE Ltd and Wiley, 2012)
